# Proteome‐Wide Profiling of Readers for DNA Modification

**DOI:** 10.1002/advs.202101426

**Published:** 2021-08-05

**Authors:** Lin Bai, Guojian Yang, Zhaoyu Qin, Jiacheng Lyu, Yunzhi Wang, Jinwen Feng, Mingwei Liu, Tongqing Gong, Xianju Li, Zhengyang Li, Jixi Li, Jun Qin, Wenjun Yang, Chen Ding

**Affiliations:** ^1^ State Key Laboratory of Genetic Engineering and Collaborative Innovation Center for Genetics and Development School of Life Sciences Institute of Biomedical Sciences Human Phenome Institute Zhongshan Hospital Fudan University Shanghai 200433 China; ^2^ State Key Laboratory of Proteomics Beijing Proteome Research Center National Center for Protein Sciences (The PHOENIX Center, Beijing) Institute of Lifeomics Beijing 102206 China; ^3^ Department of Pediatric Orthopedics Xin Hua Hospital Affiliated Shanghai Jiao Tong University School of Medicine Shanghai 200092 China

**Keywords:** DNA modification, epigenetic regulation, mass spectrometry, proteomics

## Abstract

DNA modifications, represented by 5‐methylcytosine (5mC), 5‐hydroxymethylcytosine (5hmC), 5‐formylcytosine (5fC), and 5‐carboxylcytosine (5caC), play important roles in epigenetic regulation of biological processes. The specific recognition of DNA modifications by the transcriptional protein machinery is thought to be a potential mechanism for epigenetic‐driven gene regulation, and many modified DNA‐specific binding proteins have been uncovered. However, the panoramic view of the roles of DNA modification readers at the proteome level remains largely unclear. Here, a recently developed concatenated tandem array of consensus transcription factor (TF) response elements (catTFREs) approach is employed to profile the binding activity of TFs at DNA modifications. Modified DNA‐binding activity is quantified for 1039 TFs, representing 70% of the TFs in the human genome. Additionally, the modified DNA‐binding activity of 600 TFs is monitored during the mouse brain development from the embryo to the adult stages. Readers of these DNA modifications are predicted, and the hierarchical networks between the transcriptional protein machinery and modified DNA are described. It is further demonstrated that ZNF24 and ZSCAN21 are potential readers of 5fC‐modified DNA. This study provides a landscape of TF–DNA modification interactions that can be used to elucidate the epigenetic‐related transcriptional regulation mechanisms under physiological conditions.

## Graphical abstract

Modified‐transcription factor response elements (TFREs) are developed, which profile the binding activity of TFs at DNA modifications (5mC, 5hmC, and 5fC), covering 70% of the TFs in the genome. A landscape of TF–DNA modification interaction is provided and the epigenetic‐related transcriptional regulation mechanisms are elucidated under physiological conditions. Potential readers of 5fC‐modified DNA are demonstrated and validated.

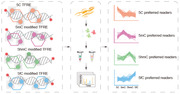

## 1 Introduction

Epigenetic modification histones and DNA play critical roles in regulating gene expression during development, differentiation, diseases, and other physiological and pathological processes.^[^ ^1^ ^]^ Methylation at the fifth position of cytosine (5mC) is the predominant epigenetic DNA modification and is highly related to the embryogenesis, development, aging, and carcinogenesis.^[^ ^2^ ^]^ The 5mC is an epigenetic marker linked to gene silencing. It is reported that the DNA methylation‐related gene downregulation results from specific interactions between transcription factors (TFs) and methylated DNA.^[^ ^1a^ ^]^ Only proteins with a methyl‐CpG binding domain (MBD) can interact with methylated DNA while the majority of TFs cannot.^[^ ^3^ ^]^ Recently, evidence of interactions between methylated DNA and TFs has emerged, such as CEBP,^[^ ^4^ ^]^ ZFHX3,^[^ ^5^ ^]^ RFX1,^[^ ^6^ ^]^ SIX4, AKSCAN3, and FOXK2,^[^ ^7^ ^]^ however, the information of the MBDs still remained unclear.

DNA methylation patterns are established and maintained by DNA methyltransferases (DNMTs).^[^ ^8^ ^]^ In 2009, the ten‐eleven translocation (TET) family dioxygenase was discovered to recognize and oxidize 5mC into 5‐hydroxymethylcytosine (5hmC).^[^ ^9^ ^]^ TET can successively oxidize 5hmC into 5‐formylcytosine (5fC) and 5‐carboxylcytosine (5caC).^[^ ^10^ ^]^ The oxidized derivatives 5fC and 5caC are recognized and excised by the mammalian thymine DNA glycosylase (TDG) and are subsequently converted to cytosine, contributing to DNA demethylation and gene regulation.^[^ ^11^ ^]^ Recent technological advances have made it possible to decode DNA methylomes and even hydroxymethylomes at single‐base pair resolution under various physiological conditions.^[^ ^12^ ^]^ DNA modifications can be screened at the genome level to assess gene expression patterns and their potential mechanisms.^[^ ^13^ ^]^ For example, while 5mC is a gene silencing epigenetic marker, hydroxylation of 5mC to 5hmC might retrieve transcription through dissociation of 5mC‐binding proteins and/or recruitment of effector proteins.^[^ ^14^ ^]^ Affinity enrichment‐based methods and modified bisulfite sequencing (BS‐seq) studies^[^ ^15^ ^]^ on pluripotent stem cells and differentiated tissues have indicated that 5hmC is obviously abundant in embryonic stem (ES) cells and neuronal Purkinje cells, and is enriched in highly transcribed gene bodies.^[^ ^16^ ^]^ With regard to the oxidized derivatives 5fC and 5caC, due to their low percentages in the genome, rare evidence of their functions on gene expression regulations has been reported. Whether 5fC and 5caC have additional DNA demethylation‐independent functions in gene regulation is not very well studied at present.

TFs, which form the second largest gene family in the genome, play critical roles in controlling gene expression patterns in almost all biological processes, including differentiation, development, cell cycle, and cell death. Approximately 1500 TF‐coding genes are annotated in the human genome.^[^ ^17^ ^]^ TFs recognized the genome sequences of the downstream genes, and the subsequently constructed the TF/transcriptional coregulator (TC)–DNA machinery. These progresses are considered the major mechanisms of gene expression regulations, in which the specific recognition of target genes (TGs) by TFs and the interactions of TF readers with these target genes are the initial steps.^[^ ^18^ ^]^ Identification of DNA modification readers, which translate modification signals into biological actions, will be crucial for deciphering the epigenetic codes of DNA modification‐mediated biological processes.

In addition to the specific binding of MBD to 5mC, the recent reports have revealed that MBD3^[^ ^19^ ^]^ and MECP2^[^ ^20^ ^]^ are able to bind to 5hmC. Furthermore, Spruijt et al. have demonstrated the specific binding of Klf4 to 5mC, and Uhrf2 to 5hmC.^[^ ^14^ ^]^ Iurlaro et al. have indicated the specific binding of RPL26 and PRP8 to 5mC and some members of the FOX family to 5fC.^[^ ^7^ ^]^ These researchers aimed to screen the potential TF “readers” of DNA modifications, especially for the 5hmC and 5fC, in order to reveal the mechanisms of the DNA modification‐related gene regulation. Although a few studies have provided many TF candidates that can interact with modified DNA in humans and mouse,^[^ ^4^, ^21^ ^]^ however, a panoramic view of the modified DNA binding activity of thousands of TFs and TCs remains elusive.

We previously developed an approach that enables the identification and quantification of the DNA‐binding activity of endogenous TFs at the proteome scale.^[^ ^22^ ^]^ With synthetic DNA containing a concatenated tandem‐array of the consensus TF response elements (catTFRE) as an affinity reagent, the TF–DNA interactions of almost all expressed TFs can be surveyed in cell lines and tissues. In this study, based on a catTFRE sequence, we prepared the 5mC‐TFRE, 5hmC‐TFRE, and 5fC‐TFRE by replacing the regular cytosine with the 5mC, 5hmC, and 5fC during the PCR amplification. The cell lines of HeLa, HepG2, A549, and MCF‐7 were used to profile the binding activity of the TFs on different DNA modifications. The binding activity on four types of DNA (5C, 5mC, 5hmC, and 5fC) was assessed for 1039 TFs, covering 70% of gene‐coding TFs, which produced the most comprehensive dataset on TF‐modified DNA interactions. Based on this dataset, the specific modified‐DNA binding TFs were identified, and the protein machineries that recognized to different DNA modifications were constructed. The different types of DNA‐binding domains (DBDs) of TFs showed diverse priorities in interacting with modified DNA. We further employed the strategy of TF‐modified DNA screening to dissect the landscape of the TF–DNA modification interactome during mouse brain development from the embryo stage to the adult stage. Finally, we validated the candidates of 5fC‐DNA‐binding TF readers, ZNF24 and ZSCAN21, and examined the relationship between epigenetics and transcriptional regulation. This study provides a rich resource that will facilitate scientists in comprehensively accessing DNA modification “readers” and elucidating the mechanisms of the gene regulation connected to the epigenetics.^[^ ^23^ ^]^

## 2 Results

### 2.1 Workflow for Preparing Different DNA‐Modification Types and the Proteome‐Wide Screening of TF‐Modified DNA Binding Activity

**Figure 1:**
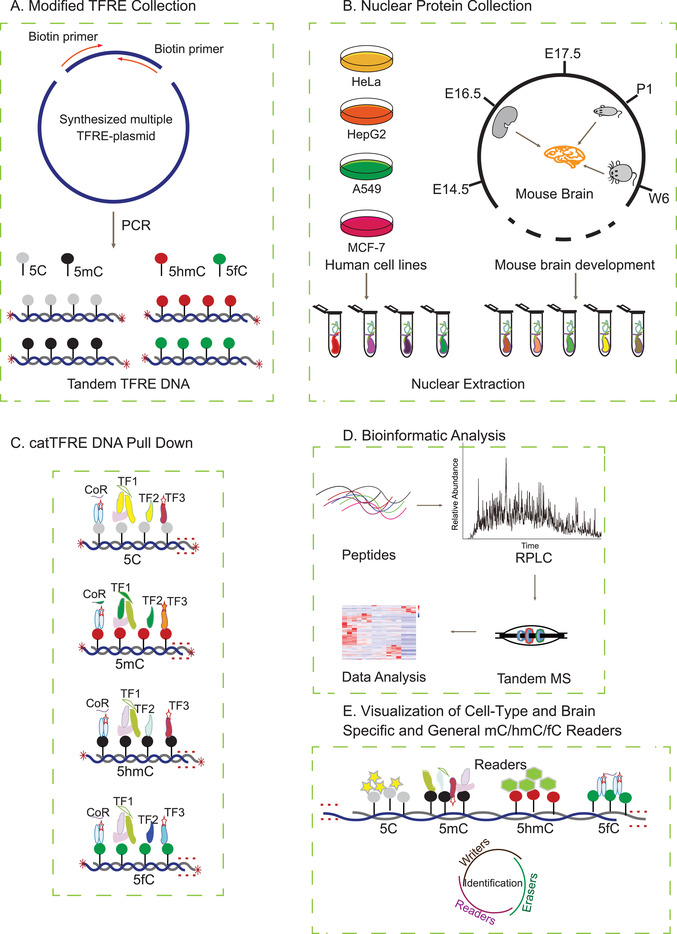
Systematic workflow for preparing different DNA modification types and proteome‐wide screening of TF‐modified DNA‐binding activity. A) Modified TFRE collection. Based on the catTFRE strategy, the four types of modified TFREs were collected using PCR technology. B) Nuclear protein collection. NEs were prepared from four human cancer cell lines and from mouse brains at different time points during development, respectively. C) catTFRE DNA pull‐down assay. The NE aliquots were incubated with the four types of modified TFREs. D) LC‐MS/MS. The general workflow for the MS‐based quantitative proteomic and bioinformatic analyses is shown. E) Visualization of cell type‐specific, brain‐specific, and general 5mC/5hmC/5fC readers.

Based on our previously developed catTFRE strategy,^[^ ^22^ ^]^ we further reformed the TFRE sequences to the 5mC‐TFRE, 5hmC‐TFRE, and 5fC‐TFRE by replacing the 5C with the 5mC, 5hmC, and 5fC, respectively, during PCR amplification (**Figure** **1**A). Consistent with 5C‐TFRE, 5mC‐TFRE, 5hmC‐TFRE, and 5fC‐TFRE have DNA lengths of 2.8 kb. To profile the modified DNA‐binding activity of TFs in humans, we made nuclear extracts (NEs) of four cell lines: HeLa, HepG2, A549, and MCF‐7. For mouse brains, the NEs were prepared at different time points during development, from embryonic day (E) E14.5 to the six weeks of age (Figure 1B). For each cell line and organ, the NE aliquots were incubated with the four types of modified TFREs (Figure 1C), and the DNA‐binding proteins were submitted to the mass spectrometry (MS) for proteome detection (Figure 1D). The proteome profiling of the four cell types were also performed for the references of the input. Bioinformatics approaches (Experimental Section) were employed to access the compositions and functions of the TF‐modified DNA interactome (Figure 1D,E).

### 2.2 Proteome‐Wide TF‐Modified DNA Binding Patterns

**Figure 2:**
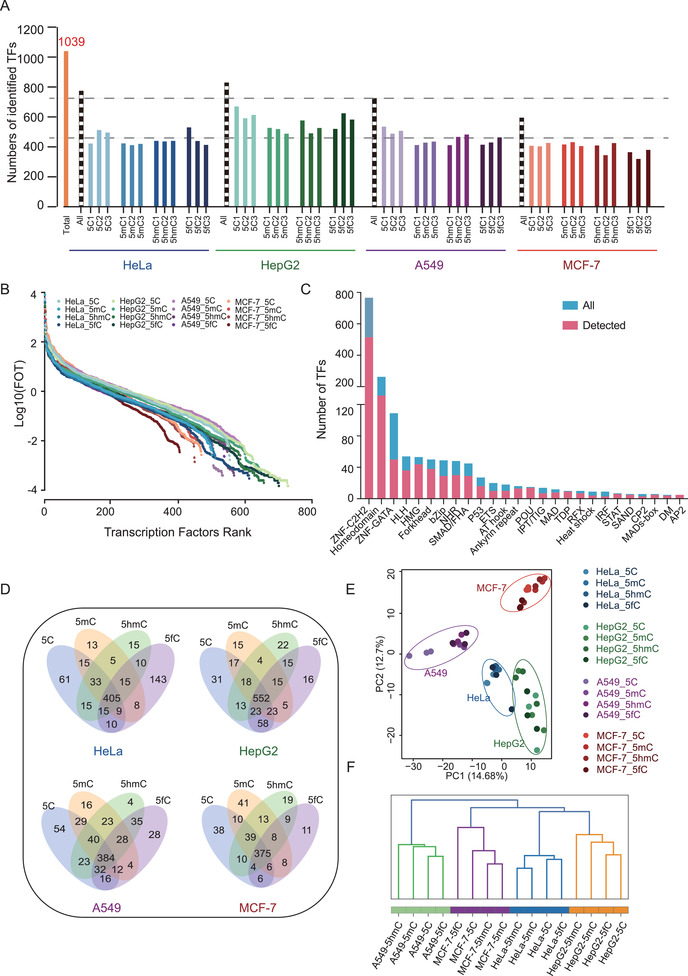
Global profiling of modified TFREs in human cancer cells. A) Total number of TFs identified in each modified‐TFRE experiment. A total of 1039 TFs were identified in modified TFREs. The numbers of identified TFs in three biological replicates for each cell line with different modified TFREs are shown, respectively. Blue indicates HeLa cell, green indicates HepG2 cell, purple indicates A549 cell, and red indicates MCF‐7 cell. The dotted line indicates the total number of TFs in four cell lines, respectively. The orange column indicates the total TF number detected in all four cell lines. B) Dynamic ranges of log_10_(FOT) in all the four cell lines measured at four different modified TFREs. C) Number of TFs identified in each TF family. A total of 26 TF families were identified. The number of TFs in each family that was detected using the catTFRE strategy is plotted. D) Venn diagrams show the overlap among the four types of modified TFRE‐interacting TFs in four cell lines, respectively. Many TFs were identified to be specific to a particular modification type. E) The dendrogram of hierarchical clustering of modified TFREs. F) PCA for the four types of modified TFREs in four cell lines. The four types of the modified TFRE were coclustered within a certain cell type rather than that of a certain modified TFRE in four types of cells.

We measured three biological replicates for each cell line with different modified TFREs. Using the TF dataset reported by Ravasi et al.,^[^ ^24^ ^]^ 400 to 700 TFs were detected in each TF‐modified TFRE experimental set, and 500–800 TFs were identified in each cell line. A total of 1039 TFs were covered in all four cell lines with the different types of modified TFREs, representing almost 70% of gene‐coding TFs in humans (the most comprehensive dataset to date for the TF and DNA modification interactome) (**Figure** **2**A). In addition to the TFs, we also have identified 480 TCs and 1805 DNA‐binding proteins (DBPs) (Figure S1A and Data S1, Supporting Information), providing a rich resource for screening of candidates of “writers,” and “erasers” in the future studies.

We employed the intensity‐based absolute quantification (iBAQ) approach for protein quantification,^[^ ^25^ ^]^ and the quantitative values were normalized with the fraction of total (FOT) value, which was each TF's iBAQ value divided by the total iBAQ value of all identified proteins. The distribution of TF abundance in each cell line covered 7 orders of magnitude, and the whole dataset ranged up to 8 orders of magnitude (Figure 2B), revealing the highly dynamic range of TF abundances. In this dataset, a total of 26 TF families were identified, and the TF families reached decent coverage. For example, 29 of 49 Forkhead family members, and 30 of 48 predicted nuclear receptors (NRs) were detected. Figure 2C summarizes the coverage for each TF family (Data S1, Supporting Information). The Venn plot indicated the different panels of TFs that were identified to interact with the different types of DNA modifications in each cell line (Figure 2D). Even though the majority of the detected TFs were overlapped in the types of DNA modification interactomes, many TFs were specific to a particular modification type. The numbers of TFs that overlapped in binding to the four types of DNA (5C, 5mC, 5hmC, and 5fC) in the four cell lines (HeLa, HepG2, A549, and MCF‐7) were, 306, 276, 268, and 276, respectively (Figure S1C, Supporting Information). Notably, the principal component analysis (PCA) showed that the TF patterns for the four types of DNA were grouped in each cell line (Figure 2E), and the hierarchical clustering demonstrated the different modified TFREs of a certain cell type were coclustered, while a certain modified TFRE was distributed in different clusters (Figure 2F), suggesting that the diversity of the cell types was greater than the diversity of the DNA modifications in a certain cell type. We further calculated the spearman's correlation coefficients for four types of cell lines and four types of DNA modifications, respectively. The results showed the correlations of different modified TFRE in a certain cell type (ranging from 0.67 to 0.89) were greater than the correlations of different cell types across four modified TFREs (ranging from 0.41 to 0.67) (Figure S1D,E, Supporting Information).

We wondered whether the diversity of the TFs in different cell types were because of their expression specificities or their modified‐DNA binding activity specificities in four cell types. To this end, we performed the Venn plot of the four cell types (Figure S2A, Supporting Information) and the four modified TFREs (Figure S2B, Supporting Information), respectively. As a result, 1039 TFs were identified, in which 412 (40%), 235 (23%), 178(17%), and 214 (21%) were identified in four, three, two, and one cell types, respectively. In the aspect of the modified TFRE, among the 1039 TFs, 726 (70%), 114 (11%), 124 (12%), and 75 (7%) were identified in the four, three, two, and one modified TFREs, respectively. The proportion of ubiquitously identified TFs in the four cell types was relatively lower than the proportion of TFs ubiquitously detected by the four modified TFREs, indicating the TF diversity might derived from their expression specificities among the four cell types. To further prove these findings, we individually assessed the overlap of the modified TFREs in a certain cell type (Figure S2C, Supporting Information), or different cell type with a certain modified TFRE (Figure S2D, Supporting Information). The proportions of identified TFs by all the four modified TFREs in certain cell type (52.3% in HeLa, 66.6% in HepG2, 52.8% in A549, and 62.7% in MCF‐7) were relatively higher than the proportions of TFs detected by certain modified TFRE in the four cell types (31.1% by 5C‐TFRE, 31.5% by 5mC‐TFRE, 30.5% by 5hmC‐TFRE, and 30.2% by 5fC‐TFRE). Besides, we further evaluated the contribution of quantitative effects to the TFs panel diversity. We calculated the coefficient of variance (CV) of TFs identified by a certain modified TFRE in all cell types, and a certain cell type by all modified TFREs, respectively (Figure S2E, Supporting Information). We found the CVs of the TFs by a certain modified TFRE in all cell types were greater than that in a certain cell type by all modified TFREs (median: 1.45 for 5C‐TFRE, 1.38 for 5mC‐TFRE, 1.35 for 5hmC‐TFRE, and 1.47 for 5fC‐TFRE; 0.91 for HeLa, 0.85 for HepG2, 0.79 for A549, and 0.44 for MCF‐7; *p*‐value < 0.0001). Together, the diversity of the TFs in different cell types might be derived from both TF expressions and modified TFRE binding activities.

We further investigated the TF‐modified DNA binding patterns for the four types of DNA in a single cell line. As shown in Figure S2F in the Supporting Information, the 5C‐DNA binding TF interactome and the 5fC‐modified DNA binding TF interactome were clearly distinguishable from each other, while the 5mC‐ and 5hmC‐modified DNA‐binding TF interactomes had a relatively high correlation. The spearman's correlation coefficients for four types of cell lines by different modified DNA TFREs also indicated a relatively close relationship between 5mC and 5hmC with a correlation ranging from 0.81 to 0.89, which were greater than that between the 5C and 5fC with a correlation ranging from 0.67 to 0.81 (Figure S1E, Supporting Information), suggesting the relative similar panel of the binding TFs between 5mC and 5hmC.

### 2.3 Functional Features of the Modified DNA Interactome

**Figure 3:**
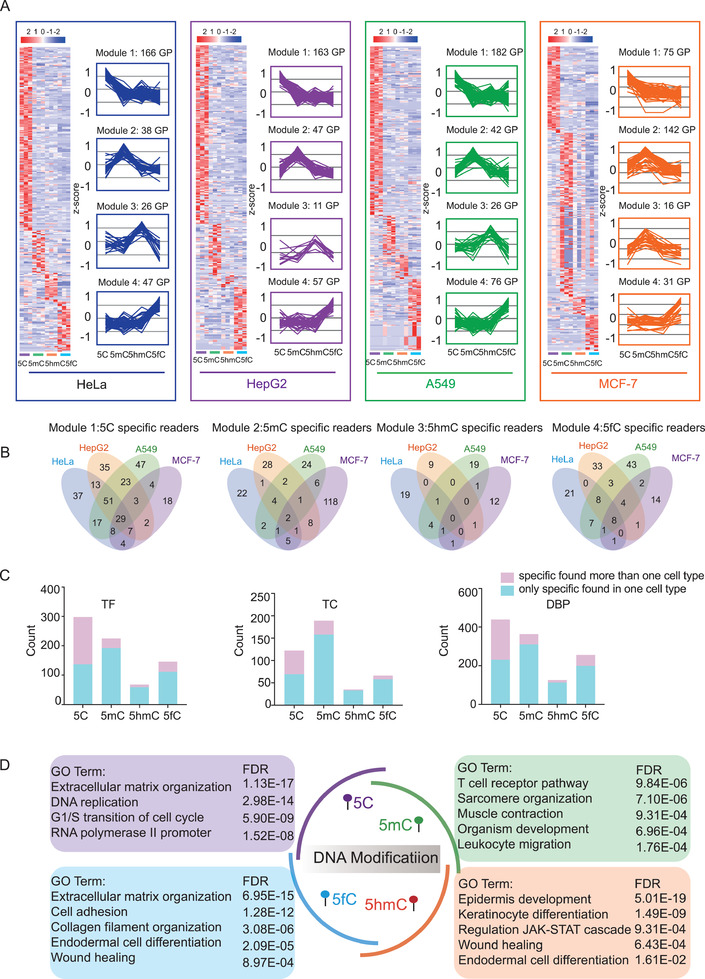
Functional features of the modified DNA interactome. A) Four dmrTF modules were revealed by cluster heatmap analysis. Left panel: Expression patterns of dmrTFs in the four modules; right panel: trend chart representative of each module. dmrTFs were defined as TFs that were identified in a DNA modification type at levels that were at least two times higher than the median value for all DNA modification types. The heatmaps and line charts summarize the DNA modification‐specific binding proteins for the four modules in each cell line. B) The Venn diagram showed the overlap of dmrTFs in four modules across four cell lines, respectively. C) The distribution of the dmrTFs, dmrTCs, and dmrDBPs that were enriched in more than one cell type (red rectangle) and specifically enriched in a certain cell type (blue rectangle) across four modified TFREs, respectively. D) GO term/pathway enrichment analysis of the dmrTFs revealed the enriched functions of the TF‐modified DNA interactomes.

We set two criteria for defining DNA modification‐restricted TFs (dmrTFs): 1) dmrTFs should have a higher binding activity for a certain DNA modification than the other DNA modifications, and 2) dmrTFs identified for a DNA modification type should exhibit levels at least twofold higher than the geometric mean value of all DNA modification types. According to these criteria, we grouped the different types of DNA specific binding proteins in each cell line into four modules, dmrTFs for 5C belonged to module 1, dmrTFs for 5mC belonged to module 2, dmrTFs for 5hmC belonged to module 3, and dmrTFs for 5fC belonged to module 4 (**Figure** **3**A). To further investigate the dmrTF patterns in different cell lines, we plotted the numbers of dmrTFs detected in the different cell lines and found that some overlapped dmrTFs across cell lines (Figure 3B). For example, ARNTL, ELF1/2/4, RXRA, RXRB, and SP3/4, among other TFs, were shown to be dmrTFs for C in all four cell lines. ZNF384 and ZHX1 showed specific binding activity for 5mC‐modified DNA in all four cell lines. ZNF618 specifically bound to 5hmC‐DNA in HeLa, A549, and MCF‐7 cell lines. ESRRB was a dmrTF for 5hmC in HeLa, HepG2, and A549 cell lines. Eight dmrTFs for 5fC‐modified DNA overlapped across all four cell lines (RFX1, RFX2, RFX3, RFX5, RFXANK, UBTF, ZFP3, and ZNF24). In addition to the overlapped dmrTFs, many dmrTFs specific to a single cell line were identified, indicating the diversity of the cell types (Data S2, Supporting Information). In the DBP and TF groups, the 5C‐DNA exhibited the greatest interaction with specific binding proteins, in which 298 dmrTFs were found to exclusively bind to 5C‐DNA. In the TC group, 5mC had the highest number of specific binding proteins, in which 189 TCs were found to specially bind to 5mC‐modified DNA (Figure 3C). Interestingly, 5hmC had the lowest number of specific binding proteins in the TF, TC, and DBP groups, while the situation was retrieved in the 5fC, suggesting the dynamic nature of the protein‐modified DNA interactions. The specific interacting TF patterns reflected the continuous transformation process of DNA modifications from 5mC to 5hmC, and 5fC: the interactomes of the 5C‐TFRE and 5fC‐TFRE showed the most significant differences, while the interactomes of 5mC and 5hmC showed gradual alterations in the middle (Figure 3A,B). We surveyed the gene ontology (GO) functions of the dmrTFs in the four cell lines (Figure S3, Supporting Information), and found consistency in the enriched TF‐modified DNA interactome features functions among cell lines. As summarized in Figure 3D, the dmrTFs for the 5C executed the ordinary functions, such as extracellular matrix (ECM)‐related function, DNA replication, ECM organization, and the cell cycle. The dmrTFs for the 5mC were enriched for endodermal cell differentiation, embryonic morphogenesis, and immune related pathways. The enriched functions of dmrTFs for 5hmC and 5fC were relatively similar, which were involved in epidermal development and cell differentiation (Data S2, Supporting Information). These findings suggested that the interaction priorities of TFs with DNA modifications were gradually switched to the embryonic‐related processes, such as DNA remodeling, cell differentiation, and tissue development, as 5C is methylated to 5mC and further oxidized to 5hmC and 5fC.

Interestingly, 136 of 298 5C‐dmrTFs, 192 of 225 5mC‐dmrTFs, 59 of 68 5hmC‐dmrTFs, and 109 of 146 5fC‐dmrTFs were exclusively found only in one cell type (Figure 3C). To investigate whether the dmrTFs that were exclusively found only in one cell type were because of their expression in different cell types, we have surveyed the identification of the dmrTFs among the four cell types. As shown in Figure S4A in the Supporting Information, 13.5–27.9% of dmrTFs were exclusively identified in one cell type, while 47.1–62.7% of the dmrTFs were found in more than three cell types. The similar phenomena were also found in the dmrTCs and dmrDBPs populations. These results indicated that the dmrTFs, which were found only in one cell type, were not because of the exclusively identification, but might be caused by the diverse modified TFRE binding activities in different cell types.

For the dmrTFs, which were exclusively determined in only one cell type (dmrTF‐EDO, exclusively determined in one cell type) by the TFRE approach, we tried to find their expression evidences in both proteome and transcriptome profiling. We performed the proteome profiling of the four cell types with the coverage of 6000–7000 proteins (Data S3, Supporting Information). Matched to the proteome profile, only few of dmrTF‐EDOs were identified in other three cell types (Figure S4B, Supporting Information). When we matched the dmrTF‐EDOs with the transcriptome data,^[^ ^26^ ^]^ an average of 18% of dmrTF‐EDOs was found in other three cell types at the mRNA level. These results demonstrated that for the dmrTF‐EDOs, which were not identified by the TFRE approach, probably do not exist in other cell types. The similar phenomena were also revealed in the DBP population, further demonstrating the sensitivity and deep coverage of the DBPs and TFs by the TFRE approach (Figure S4D, Supporting Information). Interestingly, when we surveyed the dmrTC‐EDOs population, the majority of them can be found on both the proteome and transcriptome profilings, which might be caused by the higher flexibility of the TCs in binding with the TFRE (Figure S4C, Supporting Information).

### 2.4 Weighted Gene Coexpression Network Analysis (WGCNA) Defined the DBP Modules That Were Correlated to Different DNA Modifications

To find the DBP modules that specifically bind to different modified TFREs, we then employed the WGCNA, a systems biology method for describing the correlation patterns among proteins across samples, to construct coexpression modules, which were correlated to different modified TFREs across the four cell types.

In details, 21 distinct protein coexpression modules were constructed by 1805 DNA binding proteins from the 48 datasets across the four different modified TFREs, three biological replications, and the four cell types (the parameters were TOMType = “unsigned,” corType = “unsigned,” mingene = 100, the rest was default). Spearman correlation analysis was conducted between the protein modules and the four modified TFRE (5C, 5mC, 5hmC, and 5fC) (Figure S5A, Supporting Information). As shown in Figure S5B in the Supporting Information, module purple, salmon, and green were positively correlated with 5C‐TFRE. Module brown and pink were positively correlated with 5mC‐TFRE. Module midnight blue was positively correlated with 5hmC‐TFRE. Module black, green yellow, cyan, and tan were positively correlated with 5fC‐TFRE. Furthermore, we performed GO functional enrichment analysis for the DBP modules, which were positively correlated with the four types of modified‐TFRE, respectively (Figure S5C, Supporting Information). The results showed that the protein modules bound to 5C‐TFRE were involved in regulating lipid homeostasis, histone acetylation, cell cycle, DNA replication, and so on. The protein module bound to the 5mC‐TFRE executed the cell morphogenesis involved in differentiation, fibroblast proliferation, regulation of cell cycle, etc. The enriched functions of the protein modules bound to 5hmC‐TFRE were embryonic development, endothelial cell proliferation, and so on. The protein modules bound to 5fC‐TFRE were involved in epidermis development, antigen processing and presentation, cytokinesis, and so on (Figure S5C, Supporting Information).

### 2.5 Differential Interaction Patterns of TF Families and TF Domains with DNA Modifications

**Figure 4:**
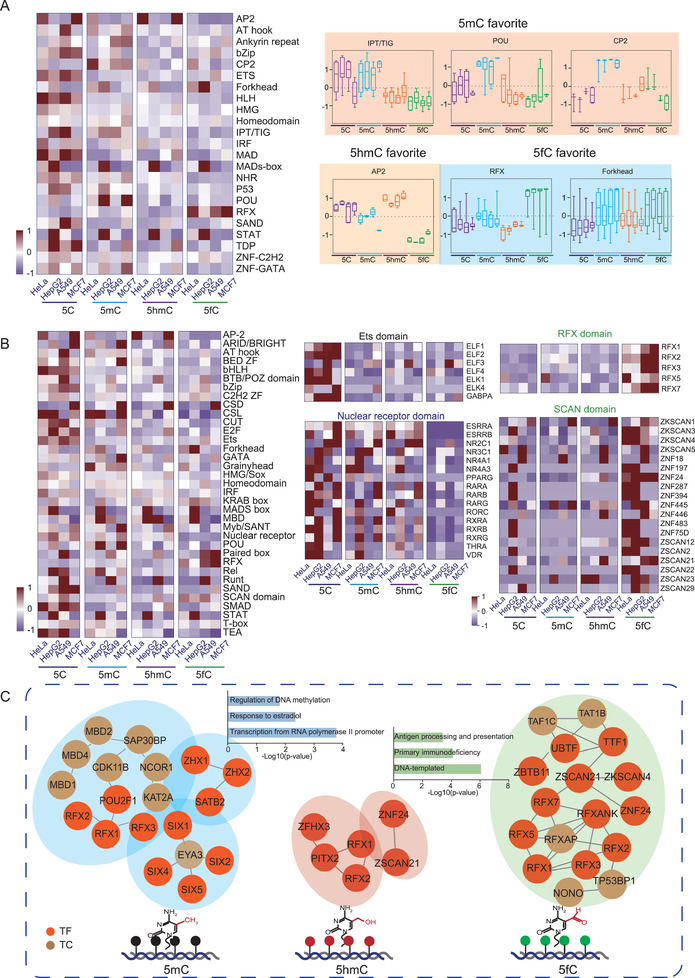
Differential combination patterns for DNA modifications with TF families and DBDs. A) Heatmap of TF DNA‐binding activity in four human cell lines with different DNA modifications. The cell lines with DNA modification were shown in the rows, and the TF families were shown in the columns. The families that preferentially bound to specific DNA modifications were shown on the right. The POU, CP2, and IPT/TIG families specifically bound to 5mC‐TFRE, while the AP2 family was specific for 5hmC‐TFRE. 5fC dominantly interacted with the Forkhead and RFX families. B) Heatmap of DBD–DNA modification interactions. The four types of DNA modifications in the different cell lines were shown in the rows, and the DBDs were shown in the columns. The detected TFs of binding domain with specific DNA modification were shown. The Ets and nuclear receptor domain preferred 5C‐TFREs. The RFX and SCAN domains recognized 5fC‐modified TFRE. C) Integration analysis of the modified DNA‐binding activity of TFs and TCs. The hierarchy of the transcriptional machineries that were specifically constructed on 5mC‐modified DNA, 5hmC‐modified DNA, and 5fC‐modified DNA, the complexes and their functional enrichment are represented by blue, pink, and green, respectively.

The TF families were basically classified according to their DBDs. As the binding of the DBDs with DNA was the structure base of the TF–DNA interactions, we then hypothesized that the TF families would have bias in interacting with different DNA modifications. In this study, we achieved deep coverage in TF family identification. Among 24 TF families, quantitative analysis showed that the majority of TF families, including the bZip, RYS, and MAD families, preferentially bound to 5C‐TFRE. However, the POU, CP2, IPT/TIG, P53, AT hook, and Forkhead families specifically bound to 5mC‐TFRE. The AP2 family was specific for 5hmC‐TFRE, while 5fC dominantly interacted with the RFX and MADs‐box families (**Figure** **4**A; Figure S6 and Data S4, Supporting Information). Such specific interactions of these TF families with different DNA modifications could explain the functions of these specific modified DNA‐binding TF families in epigenetic‐related processes, such as tissue development, cell differentiation, and carcinogenesis.^[^ ^27^ ^]^

Considering that one TF may have more than one DBD, we further assessed 35 DBDs to accurately determine the specific interactions between DBDs and DNA modifications. As shown in Figure 4B, the POU, MBD, CSD, and Grainyhead domains were specifically interacted with 5mC, the AP2 domain preferred to bind 5hmC, and the RFX and SCAN domains recognized 5fC.

We next reasoned that the real readers of the DNA modifications should exhibit the outstanding binding priorities in different cell type. To further refine the potential readers, we integrated the differential TF identification datasets and selected the candidates that showed the specific binding for DNA modifications in all four cell lines (Data S4, Supporting Information). Through this process, 24, 8, and 17 TFs were nominated as readers of 5mC, 5hmC, and 5fC, respectively. Similarly, we selected potential TC readers of the modified DNA and found 13, 1, and 8 TC readers of the 5mC, 5hmC, and 5fC, respectively.

Upon integrating the modified DNA‐binding activity of the TFs, TCs, and DBPs, we proposed a DNA modification‐specific transcriptional machinery. As shown in Figure 4C, the FOX, SIX, MBD, and SATB complexes were dominant in interacting with 5mC, the RFX complex specifically recognized 5hmC; and the RFX, SIX, and CDK complexes preferred to bind the 5fC. The hierarchy of the transcriptional machineries that are specifically constructed on modified DNA might correlate with the featured bioprocesses driven by epigenetics.

### 2.6 Dynamics of the TF‐Modified DNA Binding Patterns during Mouse Brain Development

The diverse TF binding patterns with different DNA modifications in the cell lines demonstrated that the different DNA modifications have specific transcriptional machinery‐modified DNA interactomes. During development, the mouse brain undergoes dramatic alterations in DNA modifications.^[^ ^28^ ^]^ Several lines of evidence pointed to key roles for dynamic epigenetic changes during brain development.^[^ ^29^ ^]^ We thus hypothesized that the modified DNA‐binding TFs should preferentially interact with different DNA modifications at different brain development stages. Subsequently, we investigated the dynamics of the TF‐modified DNA binding patterns during mouse brain development, assessing the roles of the differential TF‐modified DNA complexes in controlling specific gene expressions in the different stages of mouse brain development.

Brain tissue was collected at five time points during brain development, including E14.5, E16.5, E17.5, postnatal day (P)1, and week (W)6. The NEs of brain tissue at different time points were prepared and incubated with modified DNA to dissect the dynamics of the TF‐modified DNA binding patterns during brain development. A total of 435 TCs, 1182 DBPs, and 600 TFs were quantitatively detected (Figures S1B and S7A and Data S5, Supporting Information). The correlation coefficients and the heatmap suggested that the TF pattern at W6 was different from that at the other four stages from E14.5 to P1 (Figure S7B,C, Supporting Information), revealing that dramatic alterations in transcriptional regulation occur during the transition from the embryo to the adult stage. Compared to the observations in the cell lines, PCA revealed that the TF binding patterns for the four types of DNA modifications were grouped at each time point, demonstrating that diversity of the development stages was greater than the diversity of different DNA modifications at a certain development time point. Our findings indicated that despite of being separated into different brain developmental stages, 5C‐DNA binding TFs were separated from the 5mC‐, 5hmC‐, and 5fC‐modified DNA‐binding TFs at the same developmental time point (Figure S7D, Supporting Information). The Venn plot indicated the different patterns of TFs that were identified to interact with the different types of DNA modifications at each time point during brain development (Figure S7E, Supporting Information).

Figure S8A in the Supporting Information summarizes the coverage for each TF family in the mouse brain data (Data S6, Supporting Information). A total of 24 TF families were identified. Unlike in the human cell line modified DNA‐binding TF data, AP2 and DM family members were not identified in the mouse brain development. Throughout the whole mouse brain development period, the modified DNA‐binding TF family patterns at the different stages were similar (Figure S8B, Supporting Information). We also evaluated the specificities of the DBD–DNA modification interactions. Consistent with the findings obtained from the human cell line, as shown in Figure S8C in the Supporting Information, the results revealed that E2F domain specifically interacted with 5C, CP2 domain preferentially bound 5mC, and RFX domain recognized 5fC.

**Figure 5:**
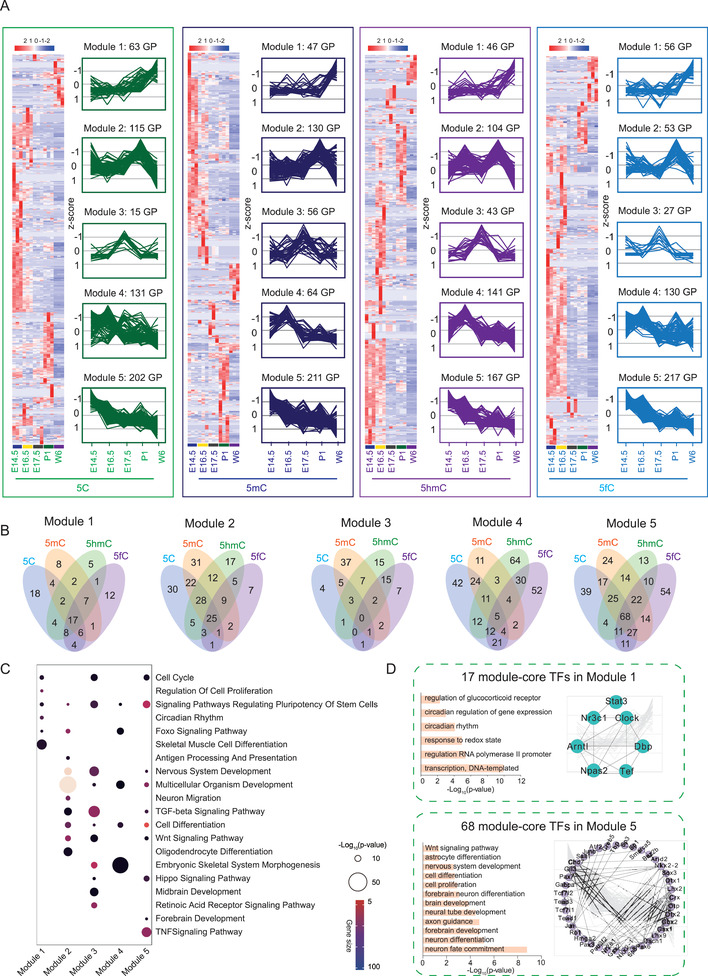
Differential interaction patterns of TF families and domains with DNA modifications. A) Specific interactions of TFs with different modified TFREs. The DNA modification‐specific binding proteins were classified into five modules. Left panel: Expression patterns of TFs in the five time points; right panel: trend chart representative of each module. B) Venn plot showed the overlap among the TFs with binding activity for the four types of modified TFREs in five modules, respectively. C) GO term/KEGG pathway enrichment analysis was investigated on all the TFs in modules 1–5, respectively. The color indicated the gene size in the GO term/KEGG term. The size of the circle indicated the *p*‐value. D) GO term/KEGG pathway enrichment analysis was investigated on the overlapped TFs binding to the four types of DNA modifications within the modules (module‐core TF), the module 1‐core TFs and module 5 TFs were constructed into network, respectively.

Based on the mouse brain development stages, we summarized the DNA modification‐specific binding proteins into five modules (**Figure** **5**A; Data S7, Supporting Information). The Venn diagram showed the different types of modified DNA binding TFs in each module (Figure 5B). We performed the GO term/KEGG pathway enrichment analysis on all TFs in modules 1–5, respectively (Figure 5C). In module 1, the TFs were enriched in circadian rhythm, cell cycle, regulation of cell proliferation and so on. In module 2, the TFs were mainly involved in regulating pluripotency of stem cells, Wnt signaling, multicellular organism development, nervous system development, and so on. In module 3, the dominant GO term/pathway were multicellular organism development, embryonic skeletal system development, regulating pluripotency of stem cells, and so on. In module 4, the TFs were significantly enriched in cell differentiation, embryonic skeletal system morphogenesis, Foxo signaling pathway, and so on. In module 5, the TFs were mainly involved in cell cycle, TNF signaling, Hippo signaling, forebrain development, and so on. The coherent expression across the different modified TFREs has revealed that the synergy synergistic and the complementary of the TFs that bound to different modified TFREs in brain development.

We further analyzed the overlap of the TFs binding to the four types of DNA modifications within the modules (module‐core TF). Interestingly, the module 1‐core TFs were enriched in the GO term/pathway of transcription, regulation of the glucocorticoid receptor, the circadian rhythm, etc. As shown in Figure 5D, Clock complexes were dominantly over‐represented with module 1. Additionally, there were more overlapped TFs (module‐core TFs) in module 5 than in the other modules. The GO term/Kyoto Encyclopedia of Genes and Genomes (KEGG) pathway analysis revealed that the module 5‐core TFs were significantly enriched in major development pathways, including nervous system development, brain development, cell proliferation, and neuron differentiation. The module 2‐core TFs were composed of Smad3, Mef2c, and Sox17. The dominant GO term/pathway were multicellular organism development, nervous system development, angiogenesis, and osteoblast development (Figure S9A, Supporting Information). The module 3‐core TFs was composed of Pou4f2, Six3, etc., and the dominant GO term/pathway were the cellular response to glucose stimulus, regulation of histone deacetylation, cell differentiation, and embryonic pattern specification (Figure S9B, Supporting Information).

The development of the mouse brain is tightly regulated by a series of signaling events and clusters of effectors in key pathways that control endoderm patterning, brain specification, brain regionalization, and morphogenesis. Changes in the protein machinery determined the fate of organogenesis and the development of the brain. The over‐represented TFs in the five time points during the mouse brain development were filtered and the TGs regulated by the TFs were surveyed according to the CellNet database. The GO term/pathway enrichment analysis of the TGs, which were regulated by the over‐represented TFs in each time point, revealed the enriched functions of the TF–DNA interactomes. As summarized in Figure S10A in the Supporting Information, the TGs at the E14.5 stage executed the functions, such as nervous system development and brain development. The TGs at the E16.5 stage were enriched in the skeletal system development, embryonic morphogenesis, vasculature development, and so on. The TGs at the E17.5 stage were enriched in secretion and differentiation, and those at P1 were enriched in cell surface receptor signaling and circulatory system development. The TGs at W6 were enriched in the cell cycle, cell death, response to DNA damage stimulus, and so on. Based on the mouse brain development data, we constructed DNA modification‐specific binding TF–TG networks. As shown in Figure S10B in the Supporting Information, the 5C‐specific TF–TG functions were mainly concentrated in the cell cycle, cell differentiation, and DNA replication categories. The 5mC‐specific TF–TG enriched functions included nervous system development, brain development, synapse organization, and so on. The 5hmC‐specific TF–TG enriched functions mainly included the BMP signaling pathway and neurotransmitter transport. Finally, the 5fC‐specific TF–TG functions were concentrated on NF‐kB signaling, inflammatory response, and apoptotic process categories, etc. The temporal dynamic TF binding activities to different DNA modifications revealed the potential mechanism of epigenetic related transcriptional regulation in the brain development.

### 2.7 Integration of Epigenetics, Proteomics, and Transcriptomics with the Modified DNA–TF Interactomes

To identify the dominant TF–TG networks, which were engaged in the DNA modification related regulation of brain development, we performed the integrated analysis of epigenetics, proteomics, and transcriptomics with the modified DNA–TF interactomes. We reasoned that the dominant TF–TG network should: 1) the TFs should specifically bind to modified TFRE and their binding activities were over‐represented in a certain time point; 2) the TGs of these TFs should be upregulated in the same time point on the mRNA level; 3) the DNA modification signals of the TGs should be detected to peaked in the same time point. To this end, we have incorporated the published paper by Ecker and co‐workers.^[^ ^30^ ^]^ They performed MethylC‐seq, TAB‐seq, and RNA‐seq on mouse brains at key developmental time points, including E14.5 and W6. The TAB‐seq represented the 5hmC level of the TGs, the MethylC‐seq represented the 5mC level of the TGs, and the RNA‐seq revealed the mRNA expression level of the TGs (Figure S11A, Supporting Information).

According to our datasets, a total of 211 and 47 TFs preferred to bind to 5mc‐TFRE in E14.5 and W6 time points, respectively. Meanwhile, 167 and 46 TFs specifically bound to 5hmc‐TFRE in E14.5 and W6 time points, respectively (Figure S11B, Supporting Information). We referred to the CellNet database for the TF–TG relationship. In the 5mC group, we surveyed the featured TGs (MethylC‐seq: *p*‐value < 0.05, fold change > 2; RNA‐seq: *p*‐value < 0.05, fold change > 2) of the 211 TFs that specifically bound to 5mC‐TFRE in the E14.5, and the 47 TFs that specifically bound to 5mC‐TFRE in the W6. As a result, 47 and 230 TGs were determined that related to the 5mC modification in the E14.5 and W6 time points, respectively. With the same criteria, 55 and 344 TGs were determined that related to the 5hmC modification in the E14.5 and W6 time points, respectively (Figure S11B, Supporting Information).

**Figure 6:**
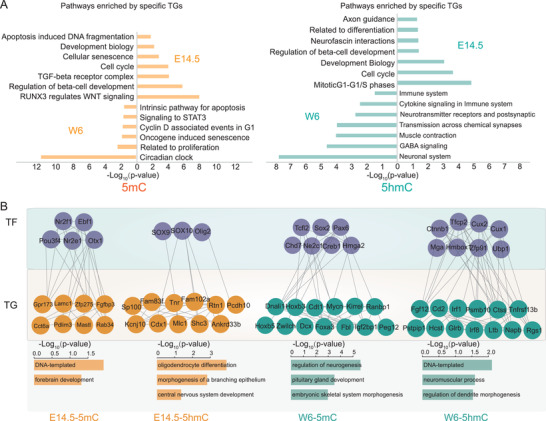
Integration of epigenetics, proteomics, and transcriptomics based on the modified DNA–TF interactome. A) GO term/KEGG pathway enrichment analysis for the TGs, which related to 5mC and 5hmC modification at E14.5 and W6 time point, respectively. B) The TF–TG networks that were dominantly related to the 5mC modification and 5hmC modification in the E14.5 and W6 time point, respectively. The first row: TFs were shown in gray color. The second row: TGs in E14.5 and W6 time points were in orange and green colors, respectively. The third row: The GO term/KEGG pathway analysis was investigated on the TF–TG network related to 5mC and 5hmC modification at E14.5 and W6 time point, respectively.

The GO term/pathway enrichment analysis demonstrated that TGs that related to 5mC modification at E14.5 were involved in the apoptosis, development, and cell cycle, while the TGs at W6 time point were related to circadian clock, proliferation, and oncogene induced senescence. Meanwhile, the TGs, which were related to 5hmC modification at E14.5 time point, were enriched at mitotic G1–G1/S phases, cell cycle, and development pathways, while the TGs, which were related to 5hmC modification at W6 time point, were enriched at neuronal system, GABA signaling, and muscle contraction biological progresses (**Figure** **6**A).

Finally, we constructed the TF–TG networks that were dominantly related to the 5mC modification and 5hmC modification in the E14.5 and W6 time points, respectively. As shown in Figure 6B, the network of TFs (Nr2f1, Ebf1, Pou3f4, etc.) and the TGs (Gpr173, Lamc1, Zfp275, Fgfbp3, etc.), participated in DNA‐templated and forebrain development pathways, was highly correlated to the 5mC modification and over‐represented in the E14.5. The network of TFs (Sox9, Sox10, and Olig2) and the TGs (Sp100, Fam83f, Tnr, Rtn1, Cdx1, etc.), related to oligodendrocyte differentiation, morphogenesis of a branching epithelium, and central nervous system development pathways, was highly correlated to the 5hmC modification and over‐represented in the E14.5. Moreover, the network of TFs (Tcfl2, Sox2, Pax6, Chd7, etc.) and the TGs (Hoxb3, Cdt1, Mycn, Kirrel, etc.), which was involved in regulation of neurogenesis, pituitary gland development, and embryonic skeletal system morphogenesis pathways, was highly correlated to the 5mC modification and over‐represented in the W6. The network of TFs (Ctnnb1, Tfcp2, Cux2, Mga, etc.) and the TGs (Fgf12, Cd2, Irf1, Psmb10, etc.), which referred to DNA‐templated, neuromuscular process, and regulation of dendrite morphogenesis pathways, was highly correlated to the 5hmC modification and over‐represented in the W6. The diversity of the TF–TG networks that dominantly correlated to different DNA modification, suggested the epigenetic transcriptional machinery functioned in the brain development.

### 2.8 The SCAN Domain‐Containing Proteins ZSCAN21 and ZNF24 Are Potential 5fC Readers

**Figure 7:**
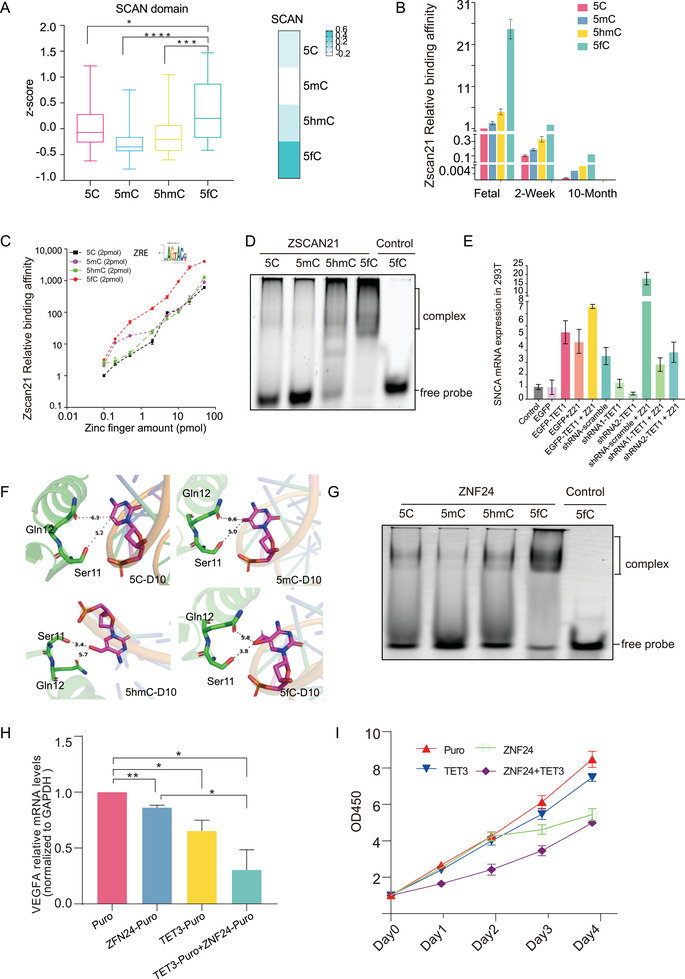
The validation of SCAN domain‐containing proteins ZSCAN21 and ZNF24 are potential 5fC readers. A) Box plot showed the SCAN domain binding activity to the four modified TFREs (pair tailed Student's *t*‐test *p*‐value < 0.05). For the box plot, the bottom and top of the box are first and third quartiles, and the band inside the box is the median of the *z*‐score. Heatmap showed the binding activity to the four modified TFREs; the darker the color, the stronger the binding activity. B) The binding priority of the endogenous ZSCAN21 in the mouse brain to 5fC‐modified TFRE based on our proteomic data. C) A series of concentrations of recombinant ZSCAN21 from 0.1 to 50 pmol were incubated with 2 pmol of the four types of modified ZREs. The MS identification and quantification have demonstrated that the 5fC‐ZRE had a higher binding affinity for ZSCAN21 than the other modified ZREs (*n* = 3 biological repeats). D) EMSA result of ZSCAN21. Four types of Cy3‐labeled DAN probes were used (5C‐(AAGTACC)_5_, 5mC‐(AAGTACC)_5_, 5hmC‐(AAGTACC)_5_, and 5fC‐(AAGTACC)_5_). A stronger mobility shift was observed in ZSCAN21/5fC‐(AAGTACC)_5_ group. E) RT‐PCR assay of SNCA level in various conditions. Overexpression of ZSCAN21 increased the expression of *SNCA*. Overexpression of TET1 also increased the expression of *SNCA*. ZSCAN21 and TET1 had synergistic effects in increasing *SNCA* abundance. F) Simulated the structure of the ZNF24–DNA binding motif complex. The gap of the ZNF24–TGCA motif interface was dramatically decreased from 5A to 3.4–3.8A when C was switched to 5hmC/5fC. G) EMSA result of ZNF24. Four types of Cy3‐labeled DNA probe were used (5C‐(TCAT)_5_, 5mC‐(TCAT)_5_, 5hmC‐TCAT)5, and 5fC‐TCAT)_5_. A stronger mobility shift was observed in ZNF24/5fC‐(TCAT)_5_ group. H) RT‐PCR assay of VEGFA level in various conditions. Overexpression of ZNF24 increased VEGFA expression. Overexpression of TET3 also increased the expression of VEGFA. ZNF24 and TET3 had synergistic effects in increasing VEGFA abundance. I) Cell proliferation assay associated with various treatments. Overexpression of ZNF24 in MCF‐7 cells inhibited proliferation; overexpression of TET3 also inhibited cell proliferation; the suppressive effect was enhanced when ZNF24 and TET3 were coexpressed.

The DBD‐modified DNA interaction analysis has revealed the priority of the SCAN domain in interacting with 5fC‐TFRE (**Figure** **7**A). This comparison strongly suggested that the TFs which containing SCAN domain^[^ ^31^ ^]^ have 5fC binding properties. We further found that the proteins ZSCAN21 and ZNF24, which contain SCAN domains, specifically interacted with the oxidized derivatives (5hmC and 5fC), especially 5fC (Figure 4B). We also confirmed that the binding priority of the endogenous ZSCAN21 for 5fC in the mouse brain, from the fetal to the adult stages (Figure 7B). To further validate the specific interaction between ZSCAN21 and 5fC‐DNA, we synthesized the ZSCAN21 binding motif AAGTACC and increased the binding affinity by 15 repeats (15×). The 15× AAGTACC was named as ZSCAN21 response element (ZRE). Similar to the modified TFREs, we prepared modified ZREs by using 5mC, 5hmC, and 5fC through PCR amplification. A series of concentrations of recombinant ZSCAN21 from 0.1 to 50 pmol were incubated with 2 pmol of the four types of modified ZREs. The MS identification and quantification have demonstrated that the 5fC‐ZRE had a higher binding affinity for ZSCAN21 than the other modified ZREs (Figure 7C). We then carried out electrophoretic mobility shift assay (EMSA) to verify whether ZSCAN21 had 5fC binding priority. Four types of Cy3‐labeled DAN probe were used (5C‐(AAGTACC)_5_, 5mC‐(AAGTACC)_5_, 5hmC‐(AAGTACC)_5_, and 5fC‐(AAGTACC)_5_). The probes were then respectively incubated with ZSCAN21 or the control protein. As shown in Figure 7D, compared to the control protein, ZSCAN21 with all the four probes showed mobility shift in gel, suggesting the DNA‐binding activity of ZSCAN21. Importantly, a significantly stronger mobility shift was observed in ZSCAN21/5fC‐(AAGTACC)_5_ group, compared to the 5C‐(AAGTACC)_5_, 5mC‐(AAGTACC)_5_, and 5hmC‐(AAGTACC)_5_ co‐incubating groups. These results indicated ZSCAN21 had the binding priority to 5fC‐(AAGTACC)_5_.

We then investigated whether the specific interaction of 5fC and ZSCAN21 was correlated with the epigenetic regulation of gene expression. The *SNCA* is the effect gene that associated with the Parkinson's disease, and a previous report has indicated that the expression of the *SNCA* is controlled by the ZSCAN21.^[^ ^32^ ^]^ Consistent with this indication, we have found a ZSCAN21 binding site in the transcription start site (TSS) region of the *SNCA*. To investigate the function of DNA‐modification in *SNCA* expression, we employed the methylcytosine dioxygenase TET1 to induce the formation of oxidized derivatives of 5mC.^[^ ^11^ ^]^ As indicated by Figure 7E, overexpression of the ZSCAN21 increased the expression of *SNCA*, confirming that *SNCA* is the downstream gene of the ZSCAN21. Overexpression of TET1 also increased the expression of *SNCA*. Importantly, the ZSCAN21 and TET1 had synergistic effects in increasing *SNCA* abundance, suggesting that ZSCAN21 acts as a potential reader of the oxidized forms of DNA modifications to regulate *SNCA* expression. This speculation was also supported by the loss‐of‐function analysis, as the knockdown of the TET1 eliminated the effect of ZSCAN21 in increasing *SNCA* levels (Figure 7E).

Our MS results also confirmed that ZNF24, which contains a SCAN domain,^[^ ^31^, ^33^ ^]^ has a strong 5fC‐modified DNA‐binding tendency. With regard to candidate 5fC readers, we found structural evidence of an interaction between ZNF24 and 5fC‐modified DNA. We simulated the structure of the ZNF24–DNA binding motif complex, and the gap of the ZNF24–TCAT motif^[^ ^34^ ^]^ interface was dramatically decreased from 5A to 3.4–3.8A when 5C was switched to 5hmC/5fC (Figure 7F). The smaller distance may have enabled the formation of hydrogen bonds to enhance the binding affinity. We also carried out EMSA to verify whether ZNF24 had higher binding activity to 5fC‐(TCAT)_5_ compared to 5C‐(TCAT)_5_, 5mC‐(TCAT)_5_, and 5hmC‐(TCAT)_5_. The synthetic 5C‐(TCAT)_5_, 5mC‐(TCAT)_5_, 5hmC‐(TCAT)_5_, and 5fC‐(TCAT)_5_, probes were respectively incubated with ZNF24 or the control protein. As shown in Figure 7G, the mobility shift of ZNF24/5fC‐(TCAT)_5_ group was significantly stronger than that of the 5C‐(TCAT)_5_, 5mC‐(TCAT)_5_, and 5hmC‐(TCAT)_5_ co‐incubating groups. These results indicated ZNF24 had the binding priority to 5fC‐(TCAT)_5_.

We hypothesized that the specific affinity of ZNF24 for 5fC‐modified DNA might be related to epigenetic gene expression. Vascular endothelial growth factor (VEGF) is a key regulator of angiogenesis,^[^ ^35^ ^]^ and previous observations have suggested that ZNF24 represses full‐length VEGF promoter activity.^[^ ^36^ ^]^ To further investigate the relative affinity of ZNF24 for modified 5fC‐modified DNA and to determine whether the interaction of 5fC‐DNA and ZNF24 was also correlated with the epigenetic regulation of gene expression, we employed the methylcytosine dioxygenase TET3 to induce the formation of oxidized derivatives of 5mC.^[^ ^11^ ^]^ As indicated by Figure 7H, overexpression of the ZNF24 decreased the expression of Vascular endothelial growth factor A (VEGFA). Moreover, overexpression of TET3 also reduced the expression of VEGFA. Importantly, the ZNF24 and TET3 had synergistic effects in decreasing VEGFA abundance, suggesting that ZNF24 also acts as a potential reader of the oxidized forms of DNA modifications to regulate VEGFA expression. To obtain insight into specific association of modifications with epigenetic regulation of gene expression, we employed a gain‐of‐function approach to assess the role of ZNF24 in MCF‐7 cell growth (Figure 7I). We constructed stable ZNF24 overexpression clones of the MCF‐7 cell line to assess the long‐term effect of ZNF24 overexpression on the growth of MCF‐7 cell. In addition, we constructed stable ZNF24 and TET3 coexpression clones of the MCF‐7 cell line. As shown in Figure 7I, compared to the control conditions, stable ZNF24 overexpression inhibited cell growth. Importantly, the ZNF24 and TET3 had synergistic effects in inhibiting cell growth. Taken together, these data suggested that stable overexpression of ZNF24 suppresses cell proliferation and that the specific interaction of 5fC and ZNF24 is correlated with the epigenetic regulation of gene expression.

## 3 Discussion

In this study, we have used quantitative MS‐based proteomics to identify readers for 5mC and its oxidized derivatives in the HepG2, A549, MCF‐7, and HeLa cell lines, as well as in fetal and adult mouse brains. Our findings provided a panoramic view of a DNA modification‐binding proteome panel covering 1400 TFs and 900 TCs. We compared our DNA modification catTFRE dataset with Iurlaro et al.’s^[^ ^7^ ^]^ and Spruijt et al.’s^[^ ^14^ ^]^ protein profiling datasets. It is clear that DNA modification catTFRE is able to capture more TFs compared with protein profiling (600 TFs in our dataset versus 112 TFs by Spruijt et al. and 67 TFs by Iurlaro et al.) (Figure S12A, Supporting Information). The 11 TFs that were exclusively in the Iurlaro et al.’s dataset observed in mouse embryonic stem cells, which were not included in our study (Figure S12B, Supporting Information). As expected, modified DNA binding activities of TFs detected by proteome profiling tend to be the high abundant ones in the DNA modification catTFRE dataset. We acknowledge that this comparison was not entirely fair as mass spectrometry technology has made great advancement. Nevertheless, comparison between the catTFRE and the profiling datasets indicated that the catTFRE could more accurately monitor the modified DNA binding activities of TFs.

Although DNA modification catTFRE is an in vitro binding method, our previous data demonstrated that our approach is able to monitor the biological response of TF dynamic changes. For instance, we utilized the catTFRE approach to analyze dynamic changes of global TF–DNA binding patterns after TNF‐*α* treatment studies.^[^ ^22^ ^]^ Afterward, we performed the catTFRE approach to measure the dynamic of TF patterns in response to EGF treatments.^[^ ^37^ ^]^ The above data indicated that the catTFRE is able to dissect cellular signaling pathways in the form of transcription factors DNA binding activity changes.

Compared to the previous findings, our results presented some DNA repair factors, such as TDG and *N*‐methylpurine DNA glycosylase (MPG). We have also identified some previously reported methylation‐binding proteins that can specifically recognize and bind to methylation sites in the MBD family (MBD1, MBD2, MBD3, MBD4, and MBD6). FOXP1, FOXP4, ESRRB, etc., which have been previously described, play roles in interpreting these epigenetic signals to enable genomic regulation. Moreover, our dataset present RFX complex members (RFX1, RFX2, RFX3, RFX5, RFXAP, and RFXANK) as potential 5fC‐modified DNA binding proteins. Spruijt et al. have demonstrated the RFX1 was identified that interact preferentially with the methylated DNA probe rather than the nonmethylated counterpart in mouse embryonic stem cell nuclear extracts.^[^ ^14^ ^]^ In another study, using a quantitative ChIP‐PCR assay, Jiang Qian et, al. demonstrated that the binding sites of RFX5 were highly methylated in the H1 human ES cell line.^[^ ^21b^ ^]^ Another study has reported that the sequence‐specific DNA‐binding protein RFX activated a methylated promoter.^[^ ^38^ ^]^ Even though RFX domain were reported to bind to the 5mC modification, the interaction of RFX domain with the 5fC modifications was not reported.

In addition to revealing the specific binding of FOXP1, FOXP4, and FOXK1, which have previously been reported to bind to 5fC‐modified DNA, we also identified that FOXJ3, ZSCAN12, and ZSCAN21 may specifically bind to 5fC‐modified DNA. The TF families were basically classified according to their DBDs. As the binding of the DBDs with DNA forms the structure basis of TF–DNA interactions, we then hypothesized that the DBDs families should have bias in interacting with different DNA modifications. We surprisingly detected the dominant binding activities of SCAN domain (ZNF24, ZSCAN21, ZSCAN22, ZKSCAN1, ZKSCAN3, ZKSCAN4, and ZKSCAN5) to the 5fC‐TFRE, suggesting the SCAN domain is a novel 5fC‐DNA reader. We then simulated the structure of the ZNF24–DNA binding motif complex, and the gap of the ZNF24–TGCA motif interface was dramatically decreased from 5A to 3.4–3.8A when 5C was switched to 5hmC/5fC. Although the function of most of the SCAN family members is unknown, an overview of selected members of this group of transcription factors suggested that the SCAN domain family is involved in the regulation of growth factor gene expression, lipid metabolism, as well as cell survival^[^ ^39^ ^]^ and differentiation.^[^ ^40^ ^]^ The interaction of SCAN domain with the 5fC suggested the above SCAN domain involved bioprocess might be regulated by the epigenetics. Collectively, the potential mechanism of both the SCAN domain and the RFX domain in the 5fC modification related epigenetics deserved further investigation.

We found a larger number (143 TFs) for 5fC‐specific TFs in HeLa cells compared to 5fC modifications in other cell types (Figure 2D). To investigate the potential reason, we analyzed the total number of TFs that preferred to bind 5fC in the four cell lines, and found that the TF identification numbers of the 5fC‐TFRE in the HeLa cell was comparable with that in other type cells (615 TFs in HeLa, 426 TFs in MCF‐7, 707 TFs in HepG2, 539 TFs in A549) (Figure S13A, Supporting Information), demonstrating the total number of TFs that bound to 5fC in the HeLa cell was not more than that in the other three cell lines. We then asked whether the 143 5fC specific binding TFs in HeLa cell were detected in the 5fC TFRE experiments in the other cell lines. As shown in Venn graph, 140 out of 143 TFs were also identified in the 5fC TFRE experiments in other three cell types, suggesting these TFs also bound to the 5fC, even though they did not show specificity in other three cell types (Figure S13B, Supporting Information). The GO term/pathway analysis of the TGs of these 143 TFs indicated that the potential of specific mechanism they functioned in the HeLa cells. Interestingly, the germ cell development, cell growth, and cell division were enriched, consistent to the biological features of HeLa cell: the progressive cell proliferation, and is correlated to the germ cell functions (Figure S13C, Supporting Information).

The quantitative nature of the DNA modification catTFRE approach allows not only confirmation of potential TF “readers” of DNA modification in a cell or tissue, but also monitoring of their dynamic change during the process of brain development. It is known that dramatic alterations in DNA modification occur during the process of brain development. Our study demonstrated that different DNA modification types have specific transcriptional machinery‐modified DNA interactomes. Our data indicated that in the E14.5 period of mouse development, the dominant pathways were the development of the brain and nervous system; in the E17.5 period, the dominant pathways were hormone secretion and the development of the immune system; and in the W6 period, the dominant pathways were the cell cycle, apoptosis, and metabolic pathways. In the five time points of mouse development, the different DNA modifications had specific transcriptional machinery‐modified DNA interactomes. The enriched GO term/pathway for 5C were the cell cycle, DNA replication and cell differentiation, while those for 5mC were the nervous system, synaptic transmission and brain development.

In a previous study, Lister et al. performed MethylC‐seq, TAB‐seq, and RNA‐seq transcriptional profiling on mouse brains at key developmental stages. This study revealed that epigenomic reconfiguration occurs during mammalian brain development. However, due to technical limitations, this study failed to identify the most of the TFs involved. After mapping this reported dataset with modified DNA–TF interactome data and transcriptomic data, we predicted the characteristic features of the 5mC and 5hmC DNA transcriptional machinery during different stages of the development. We attempted to elucidate the relationship between epigenetic inheritance and gene regulation.

Interestingly, we observed the diverse modified TFRE binding activity panels of the TFs containing the C2H2_ZF domain in human and mouse. For example, YY1, PRDM5, KLF16, KLF3, KLF7, TSHZ2, and ZBTB43 preferred to bind 5C‐TFRE in human, while their homologous gene products preferred to bind to 5hmC‐TFRE in mouse (Figure S14A, Supporting Information). We performed the GO term/pathway enrichment of the TGs of the C2H2_ZF TFs, which had diverse modified TFRE binding activities. As indicated by Figure S14B in the Supporting Information, their TGs were enriched in the different bioprocesses in human (ECM–receptor interaction, regulation of actin cytoskeleton and Wnt signaling pathway) and mouse (adherens junction, platelet activation, and TGF‐beta signaling pathway), suggesting the differences of these TFs in human and mouse. The differential modified TFRE binding activity and diverse functions of the TFs across the species deserved further investigation.

Overall, our study provides the most comprehensive DNA modification interactome, covering 70% of TFs. These datasets will allow other researchers in the field to perform in‐depth mining of different layers of epigenetic regulation. We believe that this dataset will enhance understanding of the molecular mechanisms triggered by DNA modification driven epigenetics in the crucial biological processes associated with development and disease.

## 4 Experimental Section

### Cell Culture

Human cell lines HeLa, HepG2, A549, and MCF‐7 were cultured in DMEM (Sigma) with fetal bovine serum (10%) (Invitrogen) and 1× penicillin–streptomycin (Gibco, Cat.No.15140122) in cell culture dishes (Thermo Scientific, 23017‐15) at 37 °C and 5% CO_2_.

### Animals and Tissue Collection

C57BL/6 mice (8–10‐weeks‐old) were ordered from Beijing HFK Bioscience Co., Ltd. (Beijing China) and housed under a standard specific pathogen‐free (SPF) laboratory environment. Whole brains were separated from mouse embryos during gestation or from the newborn mice after birth. Brains were collected at five time points covering the embryonic and postnatal stages: E14.5, E16.5, E17.5, P1, and W6. Each time point had three biological replicates. The whole brain tissues were washed twice with ice‐cold phosphate‐buffered saline (PBS) to remove blood and other contaminates, quick‐frozen in liquid N_2_, and stored at −80 °C for RNA or protein extractions. All animal experiments were approved by the animal care regulations of the Institutional Animal Care and Use Committee of the State Key Laboratory of Proteomics, Beijing Proteome Research Center, Beijing Institute of Radiation Medicine.

### Nuclear Extraction

Pooled brain tissues (100 mg) or cell pellets (about 200 µL volume) were washed twice with ice‐cold phosphate‐buffered saline to remove blood and other contaminates, and then suspended in cytoplasmic extraction reagent I (CER I, 800 µL) buffer (NE‐PER kit Thermo Fisher Scientific) and protease inhibitor (8 µL, Pierce, Thermo Fisher Scientific). Nuclear proteins were extracted following the protocol provided by the manufacturer. Protein concentrations were measured using Bradford method (Eppendorf Bio spectrometer). In each biological replicate, tissues from mice (3 to 4) were pooled for each sample to further minimize the individual differences between mice.

### Proteome Profiling

Protein extraction and trypsin digestion: Samples were minced and lysed in lysis buffer (8 m urea, 100 × 10^−3^ m Tris hydrochloride, pH 8.0) containing protease and phosphatase inhibitors (Thermo Scientific) followed by 1 min of sonication (3 s on and 3 s off, amplitude 25%). The lysate was centrifuged at 14 000 × *g* for 10 min and the supernatant was collected as whole tissue extract. Protein concentration was determined by Bradford protein assay. Extracts from each sample (50 µg protein) was reduced with dithiothreitol (10 × 10^−3^ m) at 56 °C for 30 min and alkylated with 10 × 10^−3^ m iodoacetamide at room temperature in the dark for additional 30 min. Samples were then digested using the filter‐aided sample preparation (FASP) method^[^ ^41^ ^]^ with trypsin. The peptide was dried in a vacuum concentrator (Thermo Scientific), and then analyzed by liquid chromatography tandem mass spectrometry (LC‐MS/MS).

### TFRE–DNA Synthesis and DNA Pull‐Downs

TF‐binding database JASPAR was referred to select consensus transcription factor response elements for different TF families. To design the catTFRE construct, 100 selected transcription factor response elements were used and two tandem copies of each sequence were placed with a spacer of three nucleotides in between, resulting in a total DNA length of 2.8 kb. Biotinylated catTFRE primers were synthesized by Genscript (Nanjing, Jiangsu Province, China).^[^ ^22^ ^]^ In this study, based on the catTFRE sequence (C‐TFRE), the 5mC‐TFRE, 5hmC‐TFRE, and 5fC‐TFRE were prepared by replacing the regular cytosine with the 5mC, 5hmC, and 5fC during the PCR amplification. In total, biotinylated DNA (3 pmol) were prebound to Dynabeads (M280 streptavidin, Thermo Fisher scientific), and then incubated with NEs in 4 °C for 2 h. After incubation, the supernatant was discarded, and the beads were washed for four times with NETN buffer (100 × 10^−3^ m NaCl, 20 × 10^−3^ m Tris‐HCl, 0.5 × 10^−3^ methylenediaminetetraacetic acid (EDTA), and 0.5%(vol/vol) Nonidet P‐40), plus one time with water. Liquid was taken out as much as possible. NH_4_HCO_3_ (50 µL) and trypsin (1.5 µg) were added, digested overnight at 37 °C with trypsin, and peptides were extracted with acetonitrile (ACN) (50%) + formic acid (FA) (0.1%), and dried under vacuum for subsequential MS analysis.

### LC‐MS/MS Analysis

Peptides from catTFRE tandem in‐solution digestion were detected by Orbitrap Fusion Lumos and Q Exactive HF (Thermo Fisher Scientific). Peptide samples were loaded onto a trap column (100 µm × 2 cm, homemade; particle size, 3 µm; pore size, 120 Å; SunChrom, USA), separated by a homemade silica microcolumn (150 µm × 30 cm, particle size, 1.9 µm; pore size, 120 Å; SunChrom, USA) with a gradient of 4–100% mobile phase B (80% acetonitrile and 0.1% formic acid) at a flow rate of 600 nL min^−1^ for 150 min. LC‐MS/MS was performed on an Orbitrap Fusion Lumos mass spectrometer using an Orbitrap mass analyzer at a mass resolution of 60 000 (Thermo Fisher Scientific, Rockford, IL, USA) coupled with an Easy‐nLC 1000 nanoflow LC system using an ion trap analyzer with the AGC target at 5e3 and maximum injection time at 35 ms (Thermo Fisher Scientific). The MS analysis was performed in a data‐dependent manner with full scans (*m*/*z* 300–1400) acquired using an Orbitrap mass analyzer at a mass resolution of 120 000 at *m*/*z* 200. The top speed data‐dependent mode was selected for fragmentation in the HCD cell at normalized collision energy of 32%, and then fragment ions were transferred into the ion trap analyzer with the AGC target at 5e3 and maximum injection time at 35 ms (Thermo Fisher Scientific), or a Q Exactive HF mass spectrometer using an Orbitrap mass analyzer at a mass resolution of 120 000 (Thermo Fisher Scientific, Rockford, IL, USA) connected to an Easy‐nLC 1000 nanoflow LC system using an Orbitrap mass analyzer at a mass resolution of 15 000 (Thermo Fisher Scientific). The MS/MS analysis was performed under a data‐dependent mode. One full scan was followed by up to 20 data‐dependent MS/MS scans with higher‐energy collision dissociation (normalized collision energy of 35%) or collision induced dissociation (normalized collision energy of 27%). Dynamic exclusion time was set with 18 s.

### Label‐Free‐Based MS Quantification for Proteins

MS raw files were processed with the Firmiana proteomics work station.^[^ ^42^ ^]^ Briefly, for mouse proteome, MS raw files were searched against the NCBI mouse Refseq protein database (released on 04‐07‐2013, 27 414 entries) in Mascot search engine (version 2.3, Matrix Science Inc.). For human proteome, MS raw files were searched against the NCBI human Refseq protein database (released on 04‐07‐2013, 32 015 entries) in Mascot search engine (version 2.3, Matrix Science Inc.).

The proteolytic cleavage sites were K, R. Up to two missed cleavages were allowed. The database searching considered cysteine carbamidomethylation as a fixed modification and *N*‐acetylation, oxidation of methionine as variable modifications. All identified peptides were quantified in Firmiana with peak areas derived from their MS1 intensity. Peptide FDR was adjusted to 1%. For protein level, the proteins that had at least one unique peptide and two high‐confidence peptides (mascot ion score > 20) were kept. For protein quantification, intensity‐based label‐free quantification, the so called iBAQ algorithm (absolute protein amounts were calculated as the sum of all peptide peak intensities divided by the number of theoretically observable tryptic peptides), was used.^[^ ^25^ ^]^ The intensity‐based absolute protein quantification of each sample was transferred into FOT (a fraction of total protein iBAQ amount per experiment), and *z*‐score was calculated using the equation *z* = (*x* − *μ*)/*σ*, (*μ* stands for the mean of the samples’ FOT, and *σ* stands for the standard deviation of the samples).

### TF Classification

Proteins identified by TFRE pull‐down were categorized into DBPs, TFs, and TCs. DBPs were extracted by filtering the genes’ description “DNA‐binding.” And, TFs and TCs were extracted by filtering the gene symbols, using the gene symbols list of TFs and TCs, from public databases described in previous studies.^[^ ^24^ ^]^

### WGCNA Analysis

WGCNA was applied to the DNA binding proteins from the 48 datasets using R code implemented in R software. The parameters were TOMType = “unsigned,” corType = “unsigned,” mingene = 100, the rest was default. Spearman correlation analysis was conducted between the protein modules and the four modified TFRE (5C, 5mC, 5hmC, and 5fC).

### RNA Extraction and Quantitative RT‐PCR Analysis

cDNAs were reverse transcribed from RNA (1.5 µg) extracted in TRIzol (Thermo Scientific) from cell cultures using the Superscript II Reverse Transcriptase (Life Technologies). Quantitative PCR was carried out on a Light Cycler 480 (Roche Diagnostics) using Fast Start SYBR Green Master (Roche Diagnostics). Quantification of gene expression was calculated as *R* = 2‐ΔΔCt, with GAPDH used as a reference gene. Primers were designed using the NCBI Primer‐BLAST software. Primer sequences are available on request.

### Cell Proliferation Assay

Cell proliferation was assessed by the Cell Counting Kit‐8 (ojindo Laboratories, Kumamoto, Japan). In brief, cells were seeded in a 96‐well plate with 4 × 10^3^ cells per well and allowed to adhere. Cell Counting Kit‐8 solution (10 µL) was added to each well, and the cells were cultured in 5% CO_2_ at 37 °C for 2 h. Cell proliferation was determined by measuring the absorbance at 450 nm.

### EMSA

The ZSCAN21 and ZNF24 were expressed and purified by *Escherichia coli* expression system. The sense strand sequences of the oligonucleotides for the EMSAs were as follows: ZSCAN21 response element, 5′‐AAGTACCAAGTACCAAGTACCAAGTACCAAGTACC‐3′, ZNF24 response element, 5′‐TCATTCATTCATTCATTCAT‐3′. Cy3‐labeled DNA probes (Cy3‐labeled 5C‐DNA, Cy3‐labeled 5mC‐DNA, Cy3‐labeled 5hmC‐DNA, and Cy3‐labeled 5fC‐DNA) were synthesis by Shanghai Generay Biotech Company. Recombinant ZNF24 (30 pmol) and ZSCAN21 (30 pmol) were incubated with four types of Cy3‐labeled DNA (3 pmol) in binding buffer containing 100 × 10^−3^ m Tris‐HCl, pH7.5, KCl (7.5 × 10^−3^ m), MgCl_2_ (25 × 10^−3^ m), NaCl (375 × 10^−3^ m), glycerol (12.5%), and DTT (5 × 10^−3^ m) at 30 °C for 30 min, respectively. DNA–protein complexes were fractionated by a polyacrylamide gel (6%) at 120 V for 35 min, at 4 °C, and visualized with a FUJIFILM FLA9000 image scanner.

### Statistical Analysis

Each experiment was performed three times. Experiment data were analyzed using GraphPad Prism 7.0 (GraphPad software, San Diego, USA) and were presented as mean values ± SD. The statistical significance was performed using unpaired Student's *t*‐test or Kruskal–Wallis test (^*^ *p* < 0.05; ^**^ *p* < 0.005; ^***^ *p* < 0.0005). Missing data were imputed using the 1/10 minimum of expression matrix, and TFRE co‐regulation network analysis was performed using the WGCNA R package to define specific modules of the four types of TFRE. Correlation was calculated by Spearman's correlation method using cor.test (Bioconductor, version 3.5.2) function in R. Both correlation coefficient and *p*‐value were computed. Hierarchical clustering analysis were implemented in python and PCA were implemented in R software. The GO terms that were enriched in the sets of enriched genes were determined using the Database for Annotation, Visualization and Integrated Discovery (DAVID) Bioinformatics Resource v 6.7 with Fisher's exact test. The functionally organized GO term network of core TFs was calculated in the STRING (version 11.0) and visualized by the software Cytoscape (version 3.6.1).

## Conflict of Interest

The authors declare no conflict of interest.

## Supporting information

Supporting Information

Supplemental Table 1

Supplemental Table 2

Supplemental Table 3

Supplemental Table 4

Supplemental Table 5

Supplemental Table 6

Supplemental Table 7

## Data Availability

All mass spectrum raw data have been deposited to iProx database. The iProx accession: IPX0002142000. The mass spectrometry proteomics data also have been deposited to the ProteomeXchange Consortium (http://proteomecentral.proteomexchange.org) via the iProX partner repository^[^ ^43^ ^]^ with the dataset identifier PXD026362. The mass spectrometry data are also available at Firmiana (http://firmiana.org/login/).
