# Supplementary material for: Proteome‐Wide Profiling of Readers for DNA Modification
Source: Adv Sci (Weinh). 2021 Aug 5;8(19):2101426. doi: 10.1002/advs.202101426 (PMC8498917; doi:10.1002/advs.202101426)
Supplement: Supplementary file 1 — Supporting Information [file ADVS-8-2101426-s007.pdf]

WILEY-VCH

© 2021 Wiley-VCH GmbH

ADVANCED SCIENCE
Open Access

Supporting Information

for *Adv. Sci.,* DOI: 10.1002/advs.202101426

Proteome-wide profiling of Readers for DNA modification

Lin Bai, Guojian Yang, Zhaoyu Qin, Jiacheng Lyu, Yunzhi Wang, Jinwen Feng, Mingwei Liu, Tongqing Gong, Xianju Li, Zhengyang Li, Jixi Li, Jun Qin, Wenjun Yang, and Chen Ding**

Figure S1

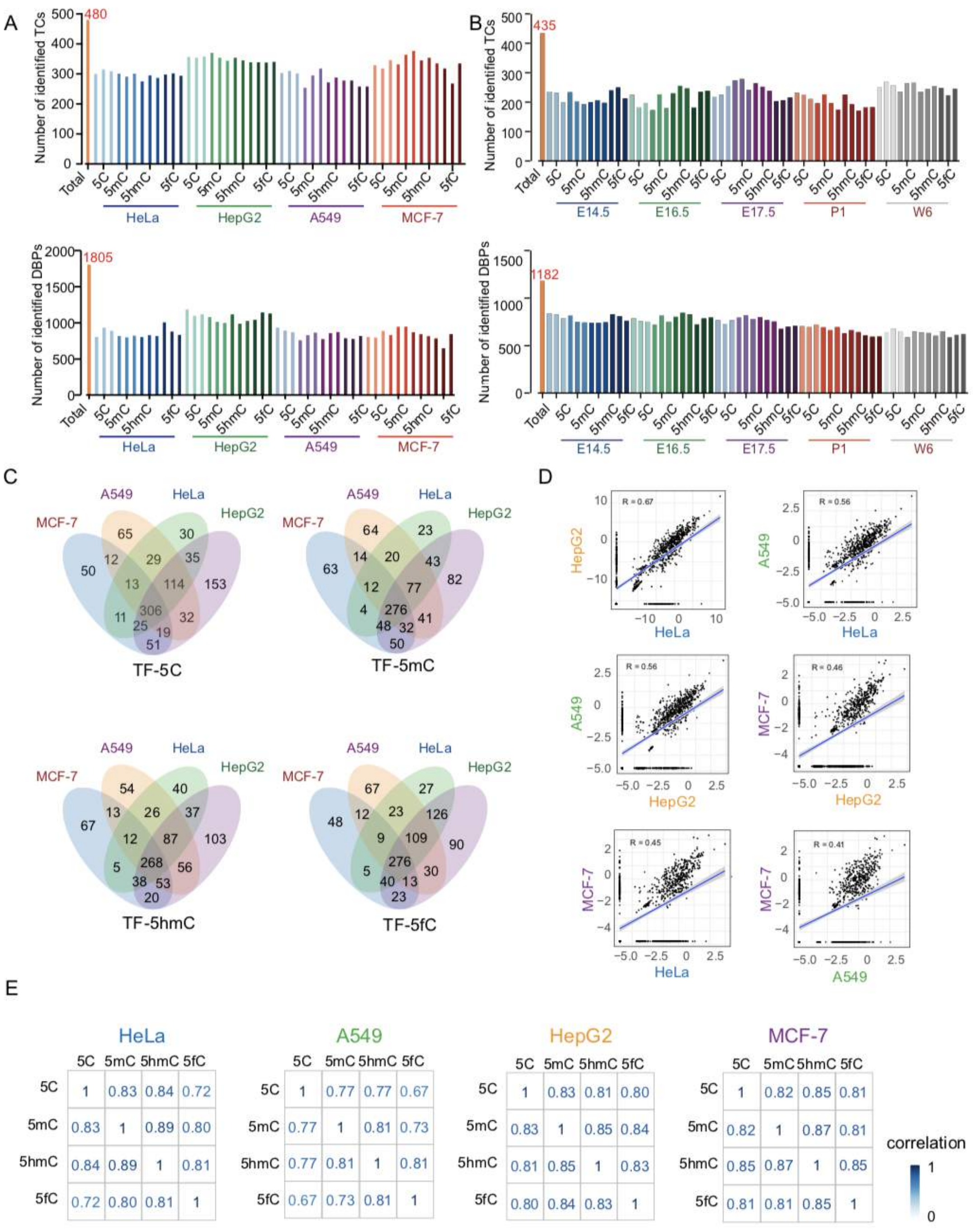

Figure S1: TCs/DBPs identified in 4 human cell lines and mouse brain. A) and B) The numbers of TCs and DBPs with different modified TFREs in each human cell line (HeLa, HepG2, A549 and MCF-7) or at different time points (E14.5, E16.5,

E17.5, P1 and E6) during the development of the mouse brain. **C**) Venn diagram showed the overlap of TFs in a certain modification between four cell types. **D**) Spearman's correlation coefficients analysis for four types of cell lines. **E**) The correlation matrix showing the correlations coefficients between the four different modified TFREs in each certain cell type.

Figure S2

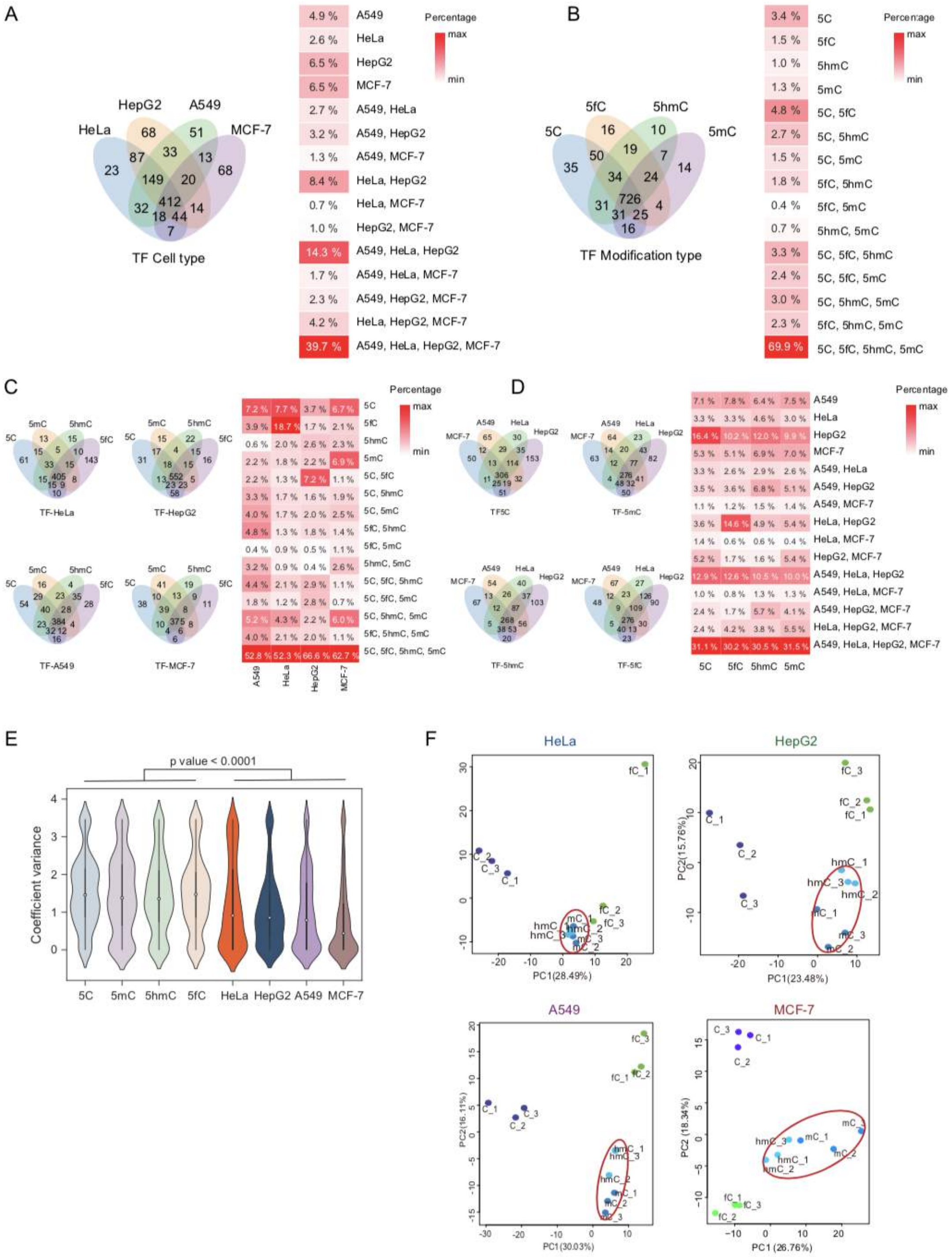

Figure S2: The diversity of qualitative effects and quantitative effects of modified TFREs. A) and B) Venn diagram showing the overlap between four cell types and four modification types, the heat map showing the fraction of each color region in each Venn diagram. **C) and D)** The distribution of the modified TFREs in certain cell

type and different cell type in certain modified TFRE, the fraction of TFs in each panel were represented in the heat map. **E)** The CVs of TFs identified by a certain modified TFRE in all cell types and a certain cell type by all modified TFREs. **F)** PCA showing the TF patterns for the four types of DNA modifications in each cell line. The 5C-modified DNA-binding activity and the 5fC-modified DNA-binding activity of TFs were clearly distinguishable from each other. The 5mC-modified DNA-binding activity and the 5hmC-modified DNA-binding activity of TFs had a relatively close relationship.

Figure S3

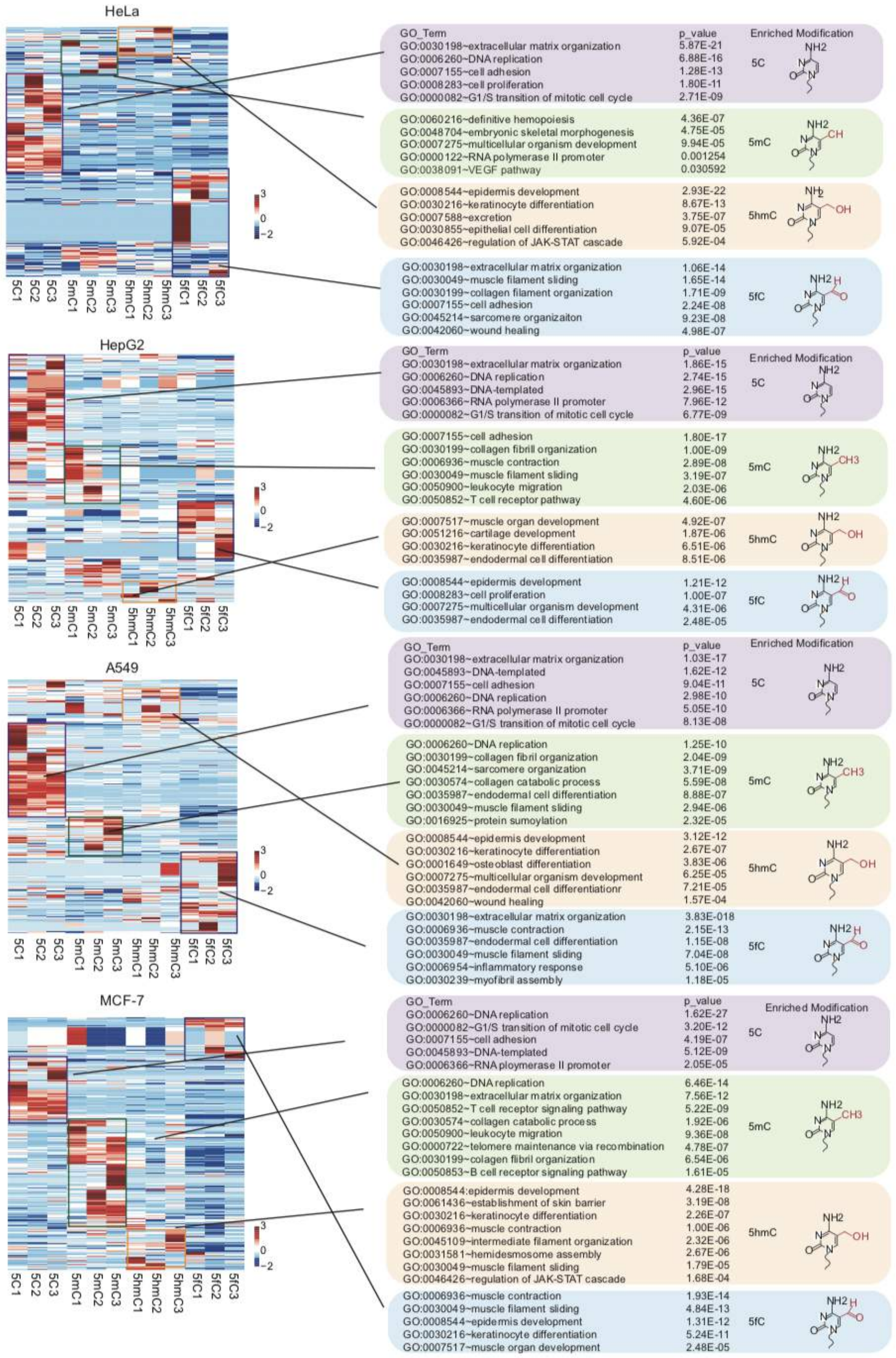

Figure S3: Heatmap of the relative abundances of DNA modifications in 3 biological replicates for each cell line with different modified TFREs. Applying a Fisher-exact-test-based enrichment to the TFs data, some gene sets from GO were

identified significantly differential enriched between four modification types in each human cell line.

Figure S4

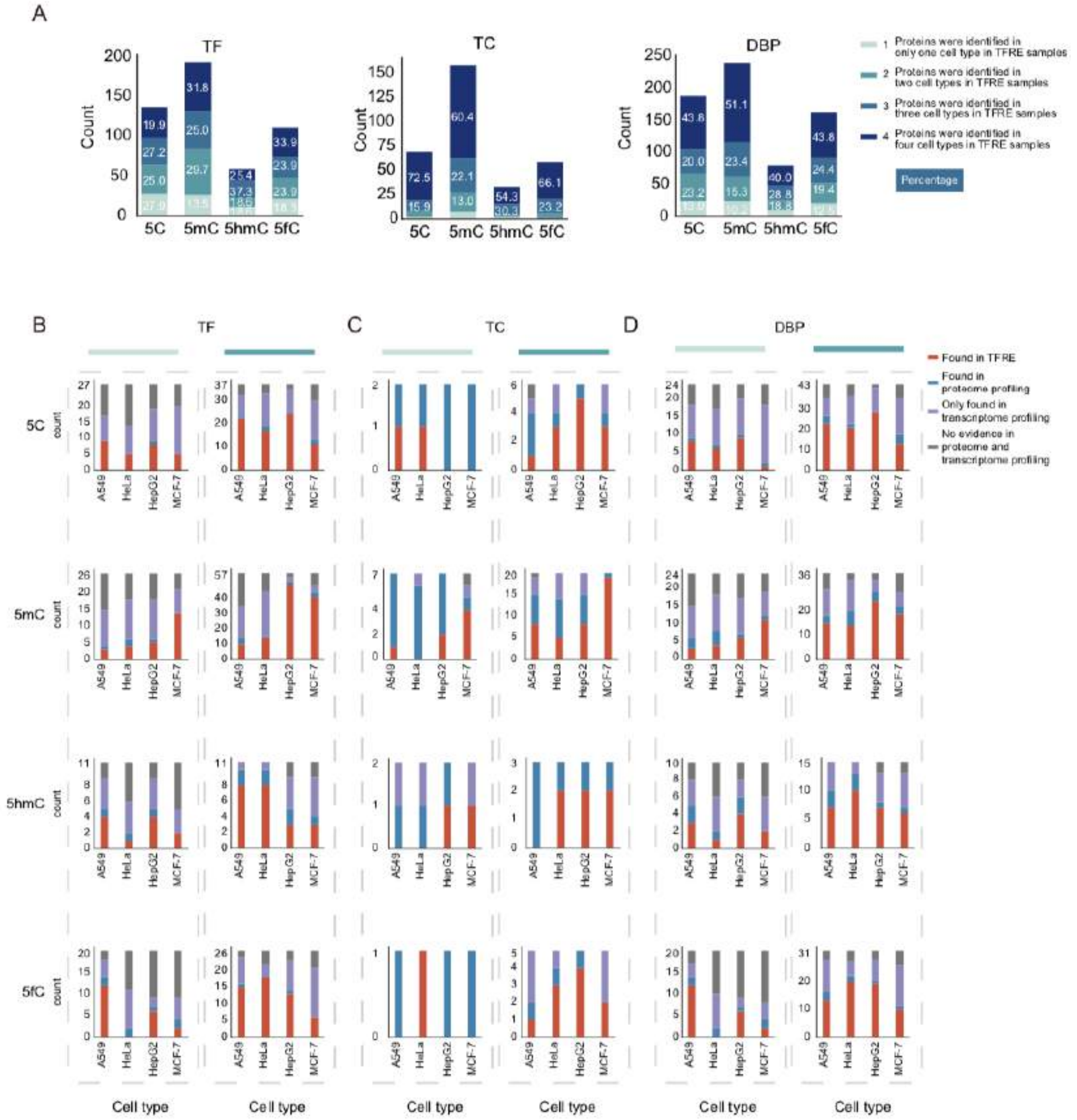

Figure S4: The distribution of dmrTFs, dmrTCs and dmrDBPs among four cell types and the evidence in proteome and transcriptional profiling. A) Histograms shows the distribution of dmrTFs, dmrTCs and dmrDBPs that were exclusively found only in one, two, three or four cell types. **B), C) and D)** The evidence of the dmrTFs/dmrTCs/dmrDBPs which specifically bound to a certain modified TFRE in proteome and transcriptome profilings. Red rectangle means the TFs/TCs/DBPs were found in modified TFRE, blue rectangle means the TFs/TCs/DBPs were found in proteome profiling, purple rectangle means the TFs/TCs/DBPs were only found in

transcriptome profiling and the grey rectangle means there was no evidence in proteome and transcriptome profilings.

Figure S5

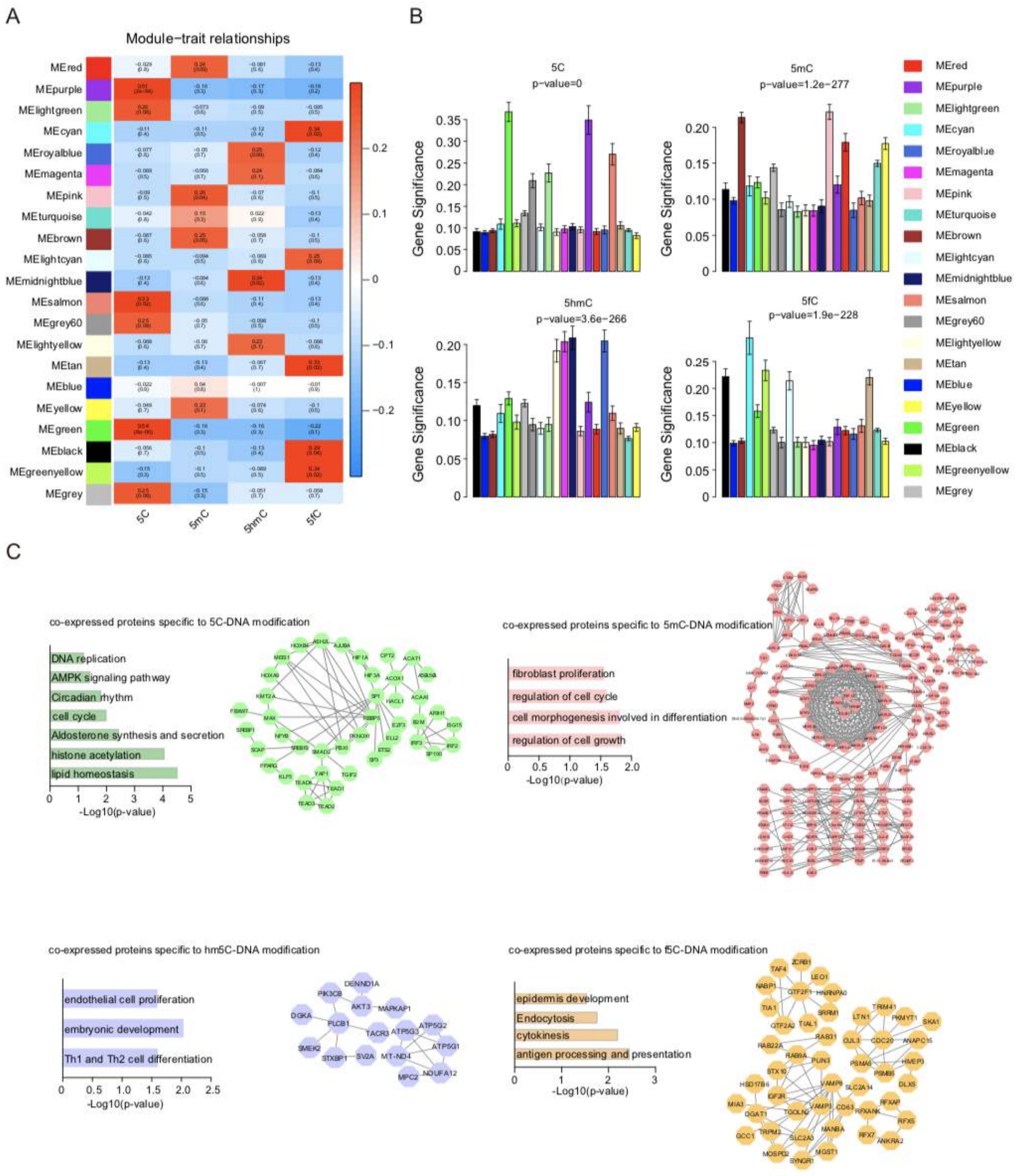

Figure S5: Weighted Gene Co-expression Network Analysis (WGCNA) and association of four modification types. A) Module-trait relationships. 21 co-expression modules were constructed and was shown in different colors. Each row corresponds to a module eigengene, column to a trait. Each cell contains the corresponding correlation and p-value. Red for positive correlation and blue for negative correlation. The table is color-coded by correlation according to the color

legend. **B)** Distribution of four modification types related DBPs in all modules. DBPs were presented in x-axis, and the enrichment significance was shown in y-axis. **C)** GO term/pathway analysis revealed the pathways that were significantly enriched in the selected modules (p-value < 0.05, correlation coefficient > 0.2) of each modification type and the network further demonstrated the TFs interactomes in the certain modification type.

Figure S6

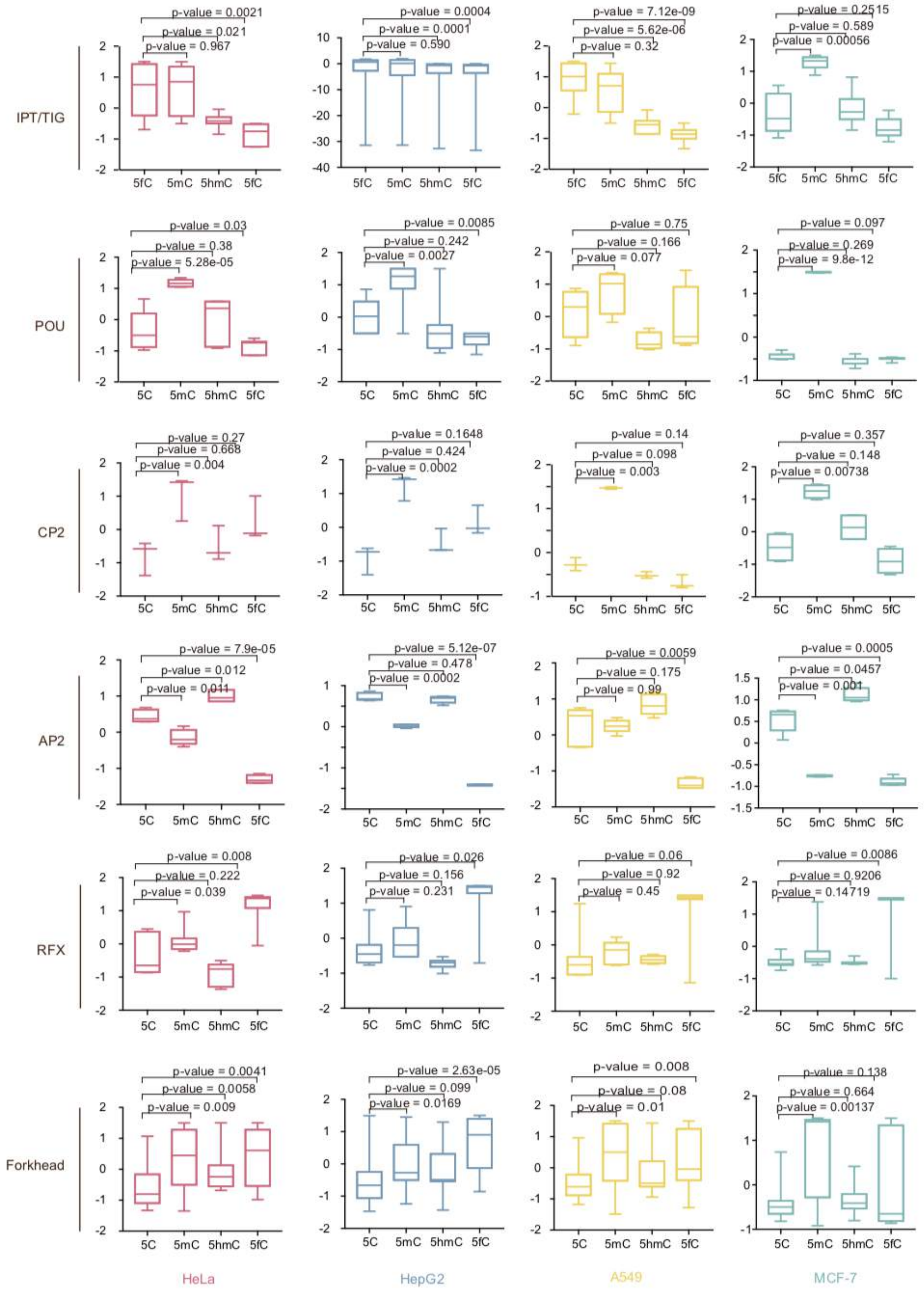

Figure S6: Box plots showed the relative abundances of the TF families specific for the different DNA modifications in four cell lines (pair tailed Student's t test $p<0.05$). The bottom and top of each box are the first and third quartiles, and the band inside the box is the median of the protein abundance.

Figure S7

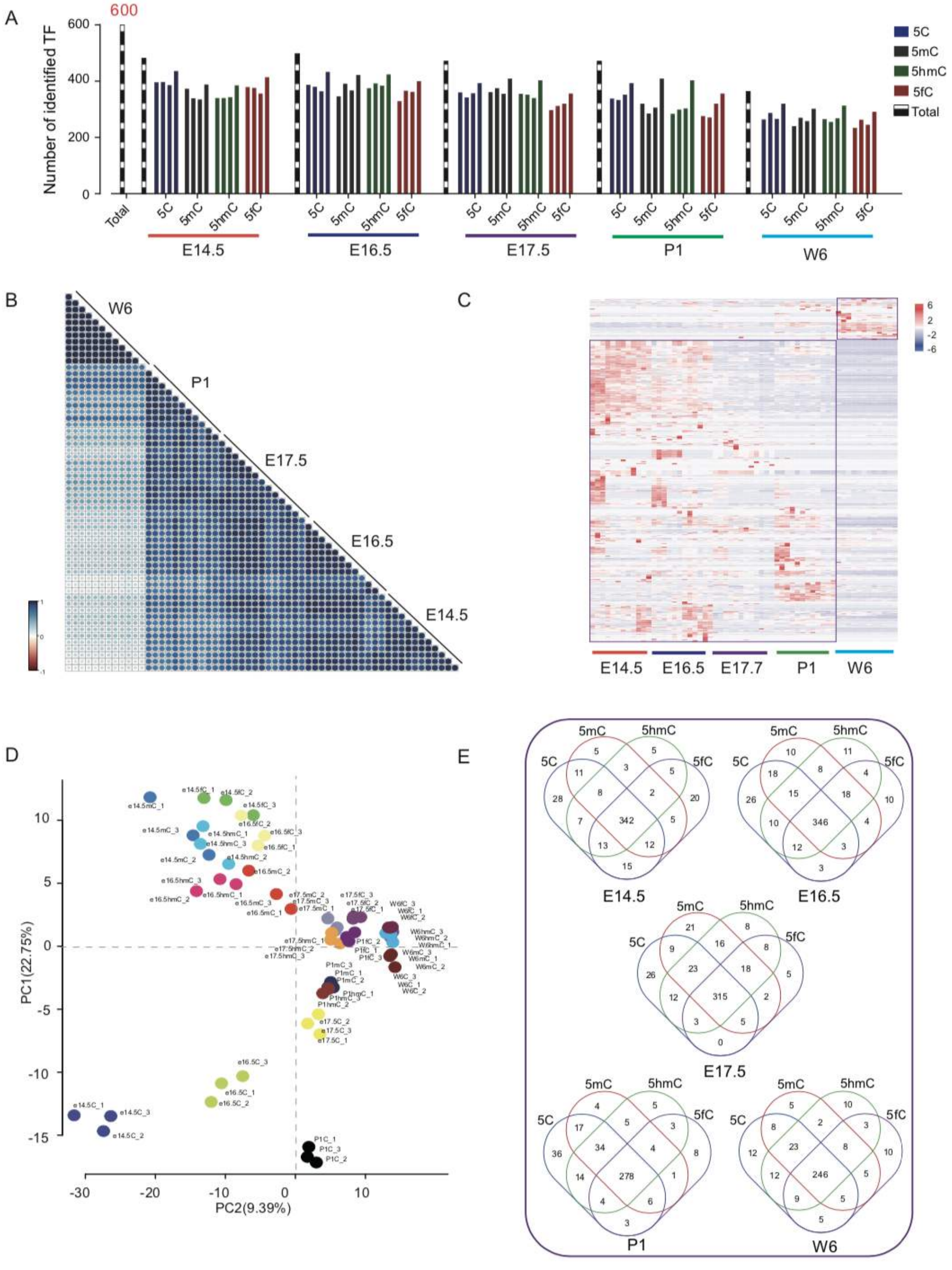

Figure S7: TFs identified at different time points during the development of the mouse brain. A) Numbers of identified TFs in 3 biological replicates for each time point during the development of the mouse brain with different modified TFREs. Red indicates E14.5, blue indicates E16.5, purple indicates E17.5, green indicates P1, and

lightblue indicatesW6 time point. The dotted line indicates the total number of TFs in five time point, respectively. A total of 600 TFs were detected in all five time points. **B)** Spearman's correlation diagram for replicate proteome profiles at different time points during mouse brain development with different DNA modifications. The degree of color in each circle represents the size of the correlation coefficient. **C)** Heatmap of the expression of TFs at different time points during development with different modified TFREs. There was an obvious difference between W6 and the other periods. **D)** PCA showing the TF patterns for DNA modifications at each time point during the development of the mouse brain. The diversity of the developmental stages was greater than the diversity of the different DNA modifications at a certain time point. **E)** Venn diagrams showing the distributions of TFs at different time points of mouse brain development with 4 types of modified TFREs.

Figure S8

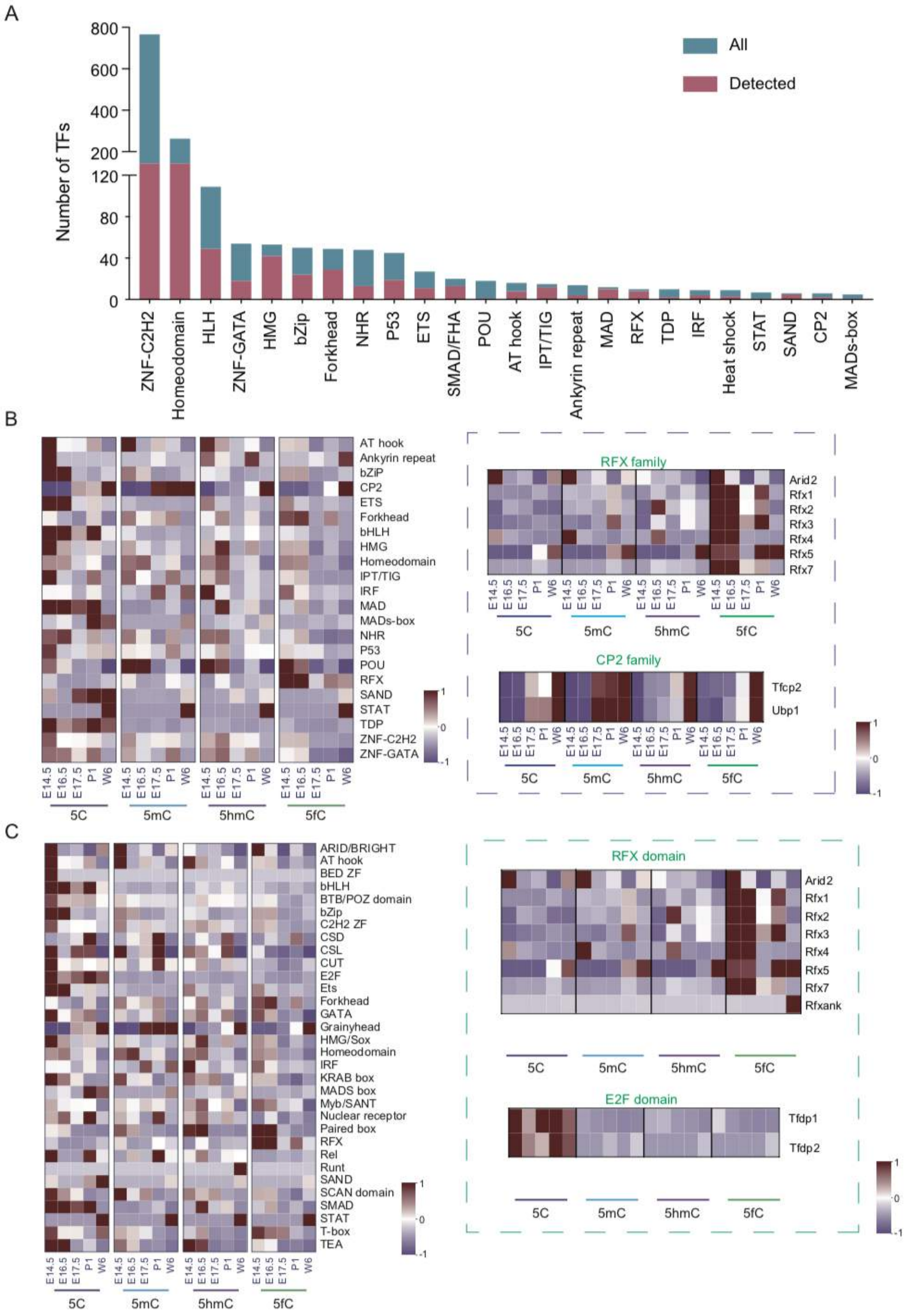

Figure S8: Coverage of TFs identified in each TF family and DNA-binding activity of TFs at different time points in the mouse brain. A) Number of TFs covered per TF family. Green represents the total number of TFs per family, and red represents the number of TFs we identified in each family. **B)** Heatmap showing the

TF DNA-binding activity at different time points during the development of the mouse brain. The time points are shown in the rows, and the TF families are shown in the columns. The TF families of potential readers (RFX family and CP2 family) for each of the modifications are shown on the right. **C)** Heatmap of DBD-DNA modification interactions. The DBDs of the detected TFs with specific affinity for DNA modifications are shown. The E2F domains was specifically interacted with 5C, while the RFX domains recognized 5fC.

Figure S9

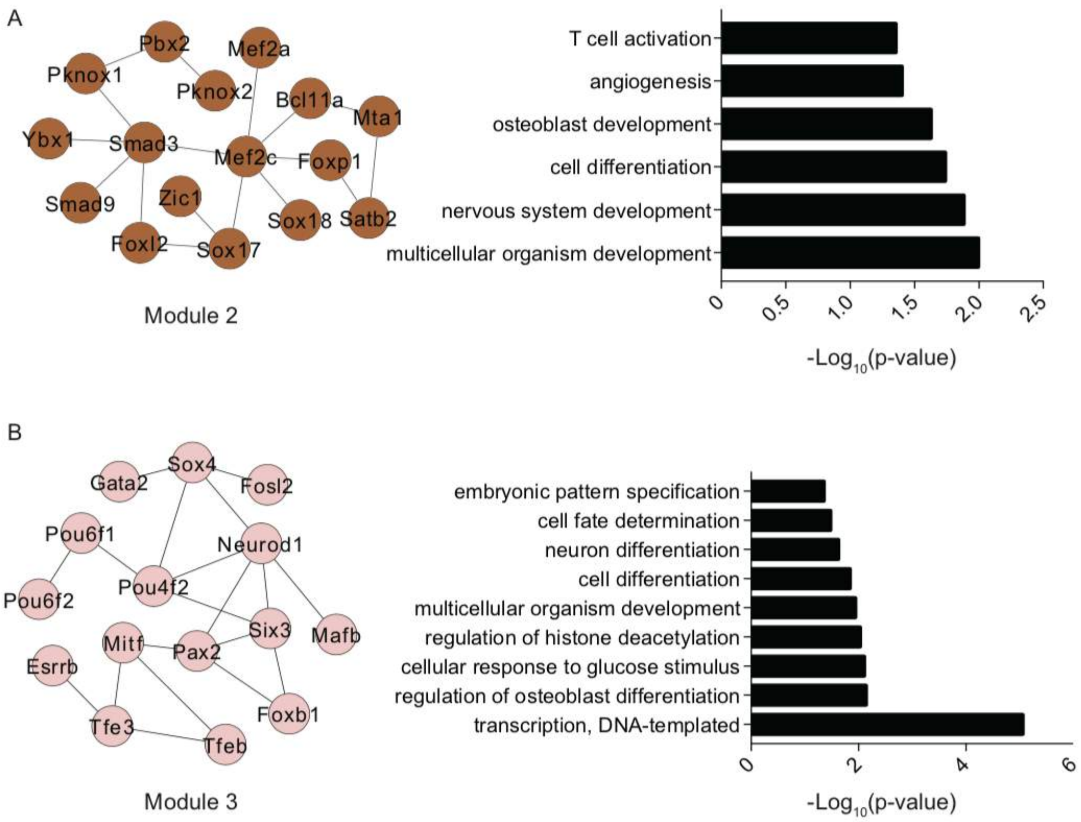

Figure S9: Construction of module core TFs network. A) and B) GO term/ pathway enrichment analysis was investigated on the overlapped TFs binding to the four types of DNA modifications within the modules (module-core TFs), the module 2-core TFs and module 3 core TFs were constructed into network, respectively.

Figure S10

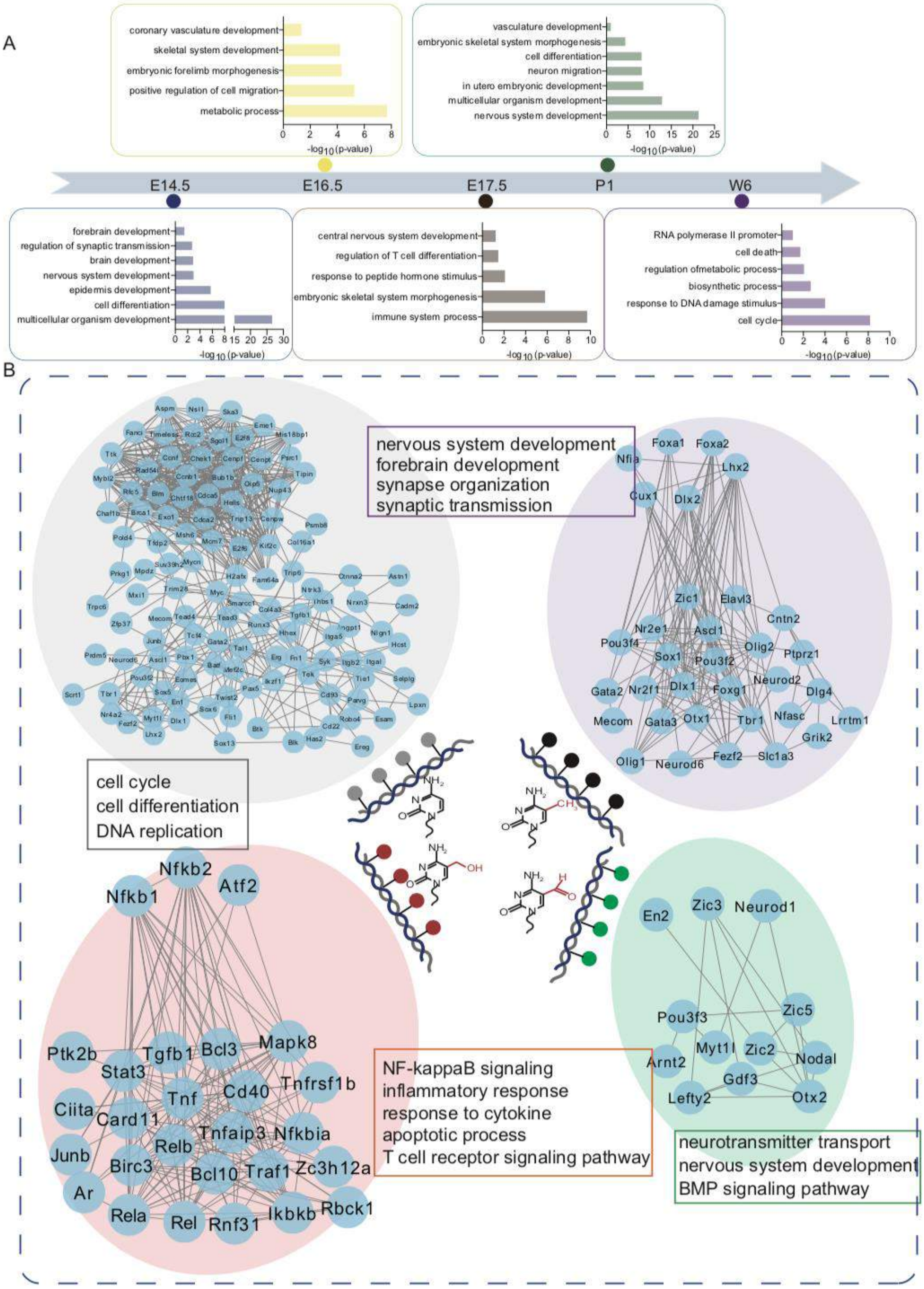

Figure S10: Different modification functional patterns A) The Functional enrichment analysis of the TGs, which were regulated by the over-represented TFs in different brain developmental stages. **B)** The networks of DNA modification-specific

binding TF-target gene showing the interactome of TF-TG in the specific modification type, further over represented analysis revealed the activated pathways in each modification type.

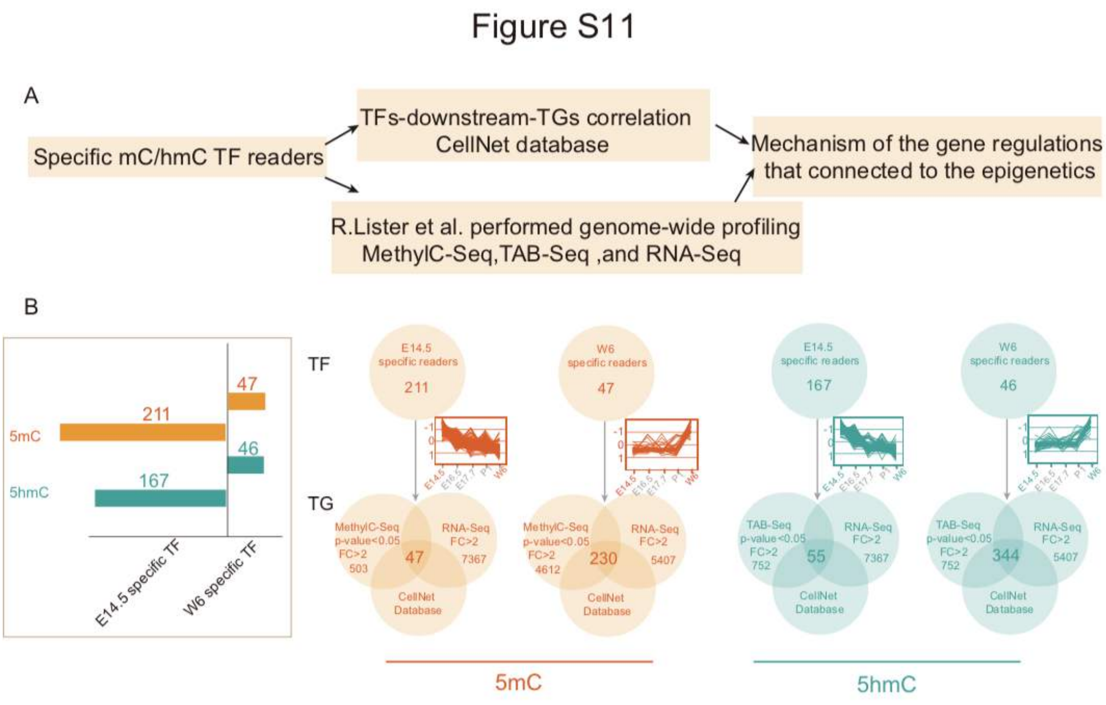

Figure S11: Integration of epigenetics, proteomics, and transcriptomics with the modified DNA-TF interactomes. A) Workflow of our attempt to elucidate the relationship between epigenetic inheritance and gene regulation using sequencing data from the literature. **B)** Number of the TFs preferred to bind to 5mC/5hmC-TFRE in E14.5 and W6 time points, respectively. Modified DNA-binding activity of TFs-TGs and specific 5mC/5hmC TF readers during mouse brain development from the *CellNet* database were integrated to build a network among TFs and TGs. In addition, sequencing data from the literature were used to elucidate the relationship between epigenetic inheritance and gene regulation.

Figure S12

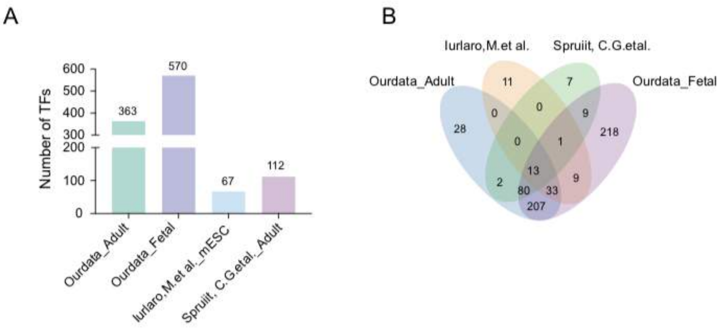

Figure S12: Comparison with the profiling data. A) Total number of the TFs identified Iurlaro, M.et al., Spruiit, C.G.et al. and our data. **B)** Venn diagram shows our data covers most of TFs that Iurlaro, M.et al. and Spruiit, C.G.et al. identified.

Figure S13

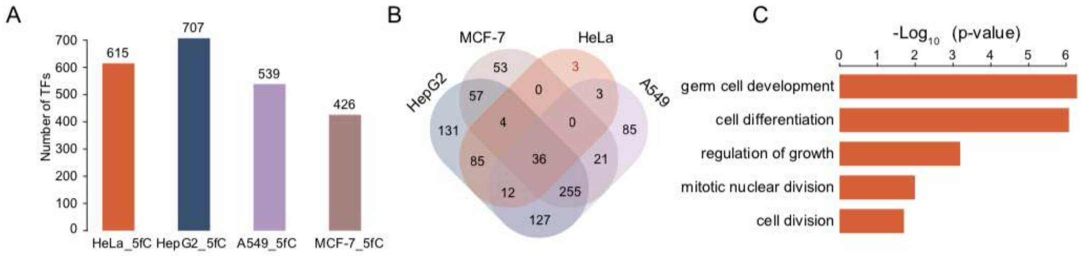

Figure S13: The 5fC-modified TFRE in HeLa cell. A) Total numbers of transcription factors that prefer to bind 5fC among the four cell lines. **B)** Venn diagram of transcription factors that only prefer to bind 5fC modification in Hela cell line and transcription factors that prefer to bind 5fC modification in other cells. **C)** The GO term/KEGG enrichment analysis of 143 proteins which appears to have preference for 5fC only in Hela cell line.

Figure S14

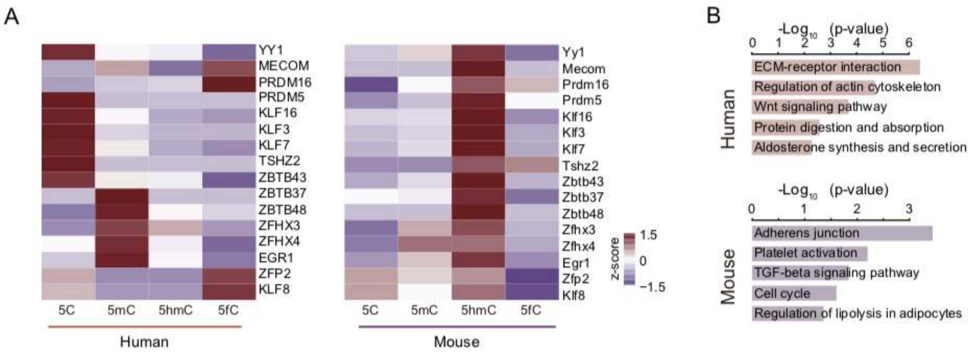

Figure S14: Comparison between human TF domain and mouse TF domain. A), TFs of C2H2_ZF co-identified in humans and mouse. **B**), GO term/KEGG pathway enrichment analysis of the co-identified TFs that containing C2H2 ZF domain in human and mouse.
